# Antidepressants act by inducing autophagy controlled by sphingomyelin–ceramide

**DOI:** 10.1038/s41380-018-0090-9

**Published:** 2018-07-23

**Authors:** Anne Gulbins, Fabian Schumacher, Katrin Anne Becker, Barbara Wilker, Matthias Soddemann, Francesco Boldrin, Christian P. Müller, Michael J. Edwards, Michael Goodman, Charles C. Caldwell, Burkhard Kleuser, Johannes Kornhuber, Ildiko Szabo, Erich Gulbins

**Affiliations:** 1 Department of Molecular Biology, University Clinic, University of Duisburg-Essen, Hufelandstrasse 55, Essen, 45122 Germany; 2 Department of Toxicology, Faculty of Mathematics and Natural Science, Institute of Nutritional Science, University of Potsdam, Nuthetal Potsdam, Germany; 3 Department of Biology, University of Padova and CNR Institute of Neurosciences, viale G. Colombo 3, Padova, 35121 Italy; 4 Department of Psychiatry and Psychotherapy, University Clinic, Friedrich-Alexander-University of Erlangen-Nuremberg, Schwabachanlage 6, Erlangen, 91054 Germany; 5 Department of Surgery, University of Cincinnati, 231 Albert Sabin Way, ML 0558, Cincinnati, OH 45229 USA

## Abstract

Major depressive disorder (MDD) is a common and severe disease characterized by mood changes, somatic alterations, and often suicide. MDD is treated with antidepressants, but the molecular mechanism of their action is unknown. We found that widely used antidepressants such as amitriptyline and fluoxetine induce autophagy in hippocampal neurons via the slow accumulation of sphingomyelin in lysosomes and Golgi membranes and of ceramide in the endoplasmic reticulum (ER). ER ceramide stimulates phosphatase 2A and thereby the autophagy proteins Ulk, Beclin, Vps34/Phosphatidylinositol 3-kinase, p62, and Lc3B. Although treatment with amitriptyline or fluoxetine requires at least 12 days to achieve sphingomyelin accumulation and the subsequent biochemical and cellular changes, direct inhibition of sphingomyelin synthases with tricyclodecan-9-yl-xanthogenate (D609) results in rapid (within 3 days) accumulation of ceramide in the ER, activation of autophagy, and reversal of biochemical and behavioral signs of stress-induced MDD. Inhibition of Beclin blocks the antidepressive effects of amitriptyline and D609 and induces cellular and behavioral changes typical of MDD. These findings identify sphingolipid-controlled autophagy as an important target for antidepressive treatment methods and provide a rationale for the development of novel antidepressants that act within a few days.

## Introduction

Major depressive disorder (MDD) is a severe and chronic disease with a lifetime prevalence of more than 10% [1]. Because suicide is the cause of death for an estimated 10% of patients with severe MDD, this disease is also often considered a potentially life-threatening illness [1]. In addition to depressed mood, MDD is characterized by a loss of interest, anhedonia, fear, feelings of worthlessness, weight loss, insomnia, and concentration deficits [1]. MDD is also associated with many somatic symptoms, including increases in the incidence of cardiovascular disease and osteoporosis, adrenocortical activation, oxidative stress levels, and plasma concentrations of proinflammatory cytokines and phospholipase A_2_, as well as decreased serum concentrations of high-density lipoprotein (HDL) cholesterol [2–7]. Despite the devastating impact of MDD, little is known about its etiology or pathophysiology.

MDD is treated with antidepressants, sleep deprivation, or electroconvulsive therapy. The previously held belief that monoamines [8] were responsible for the actions of antidepressants was recently revised because the antidepressant effect is not clearly correlated with the monoaminergic effect of these drugs; in fact, the antidepressant tianeptine is a serotonin reuptake enhancer [9]. Furthermore, the direct and rapid effect of antidepressants on the synaptic uptake of monoamines is in contrast to the delayed antidepressant effects of these drugs, which require 2–4 weeks to exhibit a clinical effect.

Thus, it has been suggested that a defect in or a reduction of neurogenesis in the hippocampus is a central element of the disease [10–13]. This notion is supported by findings showing that chronic stress and depression, in both rodents and humans, result in hippocampal atrophy [14, 15]. Two to three weeks of treatment with antidepressants induces hippocampal neurogenesis and reverses the hippocampal atrophy induced by stress, a finding consistent with the delayed action of antidepressants [10–15]. On the other hand, inhibition or ablation of neurogenesis by methods such as selective irradiation of the hippocampus does not result in MDD [11]. Most importantly, electroconvulsive therapy, an alternative treatment for MDD, and several recently developed medications, such as ketamine, produce a rapid therapeutic effect [16, 17]. The rapid therapeutic effect of these treatments is inconsistent with an extended length of neurogenesis and maturation as a requirement for antidepressive therapy. Thus, the true mechanism of action of antidepressants remains unknown.

Acid sphingomyelinase (abbreviated ASM for the human protein, abbreviated Asm for the mouse protein; EC 3.1.4.12, sphingomyelin phosphodiesterase, optimum pH 5.0; murine gene symbol, *Smpd1*) functions as a lysosomal hydrolase, catalysing the degradation of sphingomyelin to phosphorylcholine and ceramide [18]. We and others have shown that most antidepressants functionally inhibit Asm activity by inducing a partial degradation of the enzyme [19–22]. In particular, we have shown that therapeutic concentrations of the antidepressants amitriptyline and fluoxetine reduce Asm activity in the hippocampus and thereby increase neuronal proliferation, maturation, and survival and also improve behavior in models of stress-induced depression [22]. Genetic deficiency of Asm abrogates the effects of antidepressants on neurogenesis and behavior [22]. By contrast, mice overexpressing Asm exhibit constitutive behavioral changes similar to those associated with mild depression [22]. Although these studies established Asm as a target of tricyclic antidepressants, they did not identify the mechanisms by which the inhibition of Asm/ASM acts against MDD. Pharmacological inhibition or genetic deficiency of Asm results in a slow increase in lysosomal concentrations of sphingomyelin and a reduction in lysosomal concentrations of ceramide [22–24]. In particular, lysosomal concentrations of sphingomyelin change upon inhibition or deficiency of Asm. This is a slow process apparently because of compensatory mechanisms of sphingomyelin synthesis, which is controlled by the activity of the ceramide synthase pathway and sphingomyelin synthase 1 and 2 in the endoplasmic reticulum (ER) and the Golgi apparatus [25–27]. Sphingomyelin can then be transported to the plasma membrane and to lysosomes, where it accumulates slowly if it is not degraded [25–27].

In an attempt to define how antidepressants work by modifying sphingolipids, to define an overarching hypothesis of how these drugs work, and to identify new targets for the development of antidepressants, we tested whether antidepressants regulate autophagy by a modification of cellular sphingolipids.

We found that inhibiting Asm activity with 2 weeks (but not 5 days) of treatment with amitriptyline or fluoxetine results in the accumulation of sphingomyelin in lysosomes and the Golgi apparatus and, finally, in an increase in ceramide concentrations in the ER. Ceramide in the ER activates phosphatase 2A (PP2A), which mediates the activation of Ulk, followed by phosphorylation and activation of Beclin and Vps34. These kinases finally stimulate autophagy via upregulation of p62 and Lc3B. Pharmacological inhibition of sphingomyelin synthases with tricyclodecan-9-yl-xanthogenate (D609) results in very rapid (within 3 days) activation of autophagy and reverses the biochemical and behavioral signs of stress-induced MDD. In contrast, direct inhibition of Beclin with spautin-1 reduces hippocampal neurogenesis, induces behavioral changes typical of MDD, and blocks the effects of amitriptyline and D609. These studies establish a novel paradigm explaining how antidepressants act and provide a rationale for the development of novel fast-acting antidepressants.

## Results

### Antidepressants increase sphingomyelin concentrations in lysosomes and the Golgi apparatus and increase ceramide concentrations in the ER

**Fig. 1:**
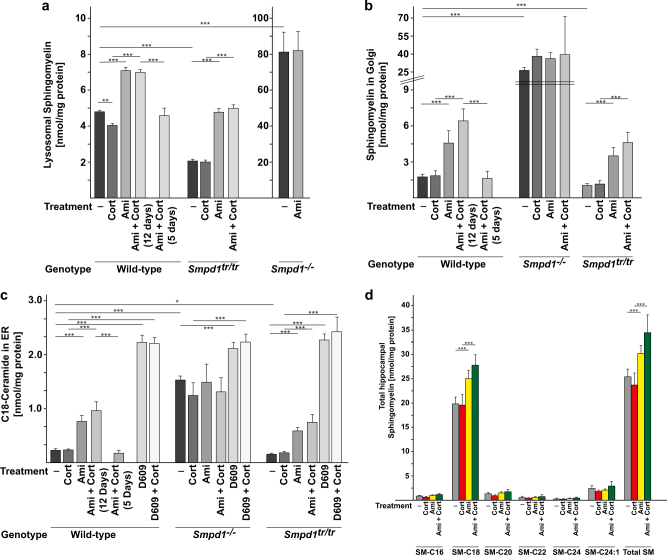
Tricyclic antidepressants induce an increase in sphingomyelin and ceramide concentrations in the Golgi and endoplasmic reticulum by inhibiting acid sphingomyelinase activity. Treating wild-type (wt) mice or acid sphingomyelinase (Asm)-overexpressing transgenic mice (*Smpd1*^*tr/tr*^) with amitriptyline (Ami) in the presence or absence of corticosterone (Cort) stress results in a time-dependent increase in lysosomal (**a**) and Golgi body (**b**) sphingomyelin concentrations and an increase in ceramide (**c**) concentrations in the endoplasmic reticulum (ER) isolated from the hippocampus. Genetic deficiency of Asm (*Smpd1*^*-/-*^-mice) abrogates all effects of antidepressants (**a**–**c**). D609, an inhibitor of sphingomyelin synthases, increases ceramide concentrations in the ER of the three mouse strains, independent of Asm expression (**c**). Sphingomyelin and ceramide concentrations were measured by mass spectrometry in isolated lysosomes (**a**), Golgi bodies (**b**), and the ER (**c**) from the hippocampus of wt, Asm-transgenic, and Asm-deficient mice. **d** Antidepressants induce an increase in the concentrations of all species of sphingomyelin in the hippocampus of wt mice, but predominantly an increase in concentrations of C18 sphingomyelin. Sphingomyelin concentrations were measured in extracts from the hippocampus of wt mice treated as indicated. Accordingly, C18 ceramide accumulates in the ER after treatment with amitriptyline (**c**). Amitriptyline was applied for 12 days if not otherwise indicated. Shown are means ± SD; *n* = 5 each; **p < 0.01, ***p < 0.001, ANOVA

Tricyclic antidepressants have previously been shown to target Asm [22]. To elucidate how inhibition of the enzyme’s activity affects MDD, we investigated changes in cellular sphingomyelin concentrations as a potential mediator of the antidepressant effects of a pharmacological inhibition of Asm. To this end, we isolated lysosomes from the hippocampus of wild-type (wt) mice that were (1) stressed with corticosterone, (2) treated with amitriptyline, (3) stressed with corticosterone and treated with amitriptyline for 5 or 12 days, or (4) left untreated (Fig. 1a and Supplementary Fig. 1). The results of mass spectrometry analysis indicated that treating mice with amitriptyline induced an accumulation of lysosomal sphingomyelin after 12 days, but not after 5 days (Fig. 1a). Stress alone slightly reduced lysosomal sphingomyelin concentrations.

Sphingomyelin is synthesized from ceramide in the Golgi bodies by the activity of sphingomyelin synthases and is then transported to the plasma membrane and lysosomes [25–27]. We therefore investigated whether the increase in lysosomal concentrations of sphingomyelin also translates into an increase in the concentrations of sphingomyelin and ceramide in the Golgi bodies and the ER. Mass spectrometry analyses of Golgi bodies and ER isolated from the hippocampus showed that treating mice with amitriptyline in the presence or absence of corticosterone stress increased the concentrations of sphingomyelin in the Golgi bodies (Fig. 1b) and of ceramide in the ER (Fig. 1c). Oral application of amitriptyline required 12 days to induce changes in the concentrations of sphingomyelin and ceramide in Golgi bodies and the ER (Fig. 1b, c) but had no effect after 5 days, a finding consistent with the changes in lysosomes of cells treated with amitriptyline (Fig. 1a).

Next, we investigated whether bypassing lysosomal metabolism by direct inhibition of sphingomyelin synthases with the inhibitor of D609 [28, 29] also results in rapid accumulation of ceramide in the ER. The results show that treatment of unstressed or corticosterone-stressed wt mice with D609 increased ceramide concentrations within the ER after only 3 days (Fig. 1c), a finding that is in marked contrast to the slower action of amitriptyline. Approximately 90% of all ceramide in hippocampal extracts from the ER was C_18_-ceramide; the percentages of other species were close to the detection limit and are therefore not reported. The results from control mice demonstrated that treatment with D609 does not inhibit Asm activity in the hippocampus and does not change lysosomal sphingomyelin concentrations in isolated hippocampal lysosomes, at least at the doses used in the present study (Supplementary Fig. 2).

The increase in cellular concentrations of sphingomyelin upon treatment of unstressed or stressed mice with amitriptyline was also detected in total extracts of the hippocampus of these mice (Fig. 1d). Analysis of the various sphingomyelin species showed that predominantly C_18_-sphingomyelin concentrations increased after treatment with amitriptyline (Fig. 1d).

Western blots of purified lysosomes, Golgi bodies, and the ER, as detected by antibodies against Lamp1 (a lysosomal marker), calreticulin (an ER marker), Golga1 (a Golgi body marker), VDAC1 (a mitochondrial marker), or S6K (a cytosol marker), confirmed purification of the organelles (Supplementary Fig. 3).

We have previously shown that antidepressants target Asm [22, 30]. Therefore, using Asm-deficient and Asm-transgenic mice, we investigated whether the change in lysosomal sphingomyelin concentrations after 12 days of treatment with amitriptyline is caused by a specific inhibition of Asm.

Mass spectrometry studies showed that mice lacking Asm exhibit increased levels of sphingomyelin in lysosomes but only a slight increase in ceramide levels in the ER, a finding indicating a compensatory mechanism upon Asm deficiency (Fig. 1a–c). Treating stressed or unstressed Asm-deficient mice with amitriptyline did not change these levels, whereas treating Asm-deficient mice with D609 increased the concentration of ceramide in the ER (Fig. 1c). Analysis of Asm-transgenic mice, which exhibit an approximately fivefold higher activity of Asm in the hippocampus [22, 30], showed a reduction of sphingomyelin concentrations in lysosomes and the Golgi and of ceramide concentrations in the ER (Fig. 1a–c). Treating unstressed or stressed Asm-overexpressing mice with amitriptyline increased sphingomyelin concentrations in lysosomes and the Golgi and ceramide concentrations in the ER (Fig. 1a–c). Furthermore, blocking sphingomyelin synthases with D609 also increased ceramide concentrations in the ER of these mice (Fig. 1c).

To confirm the results of these mass spectrometry studies and, in addition, to study also the effect of fluoxetine on ER ceramide concentrations, we performed ceramide kinase assays with isolated ER from the hippocampus of wt or Asm-deficient mice treated with fluoxetine for 5 or 12 days with or without corticosterone or left untreated. The results showed an increase in ceramide concentrations in the ER after treatment with fluoxetine for 12 days (Supplementary Fig. 4), a change that is similar to the increase in ceramide concentrations in the ER after treatment with amitriptyline. Treatment with fluoxetine for 5 days failed to alter ceramide in the ER. Fluoxetine did not change ceramide levels in the ER of hippocampus cells in Asm-deficient mice.

### Antidepressants and D609 induce autophagy and formation of autophagolysosomes

**Fig. 2:**
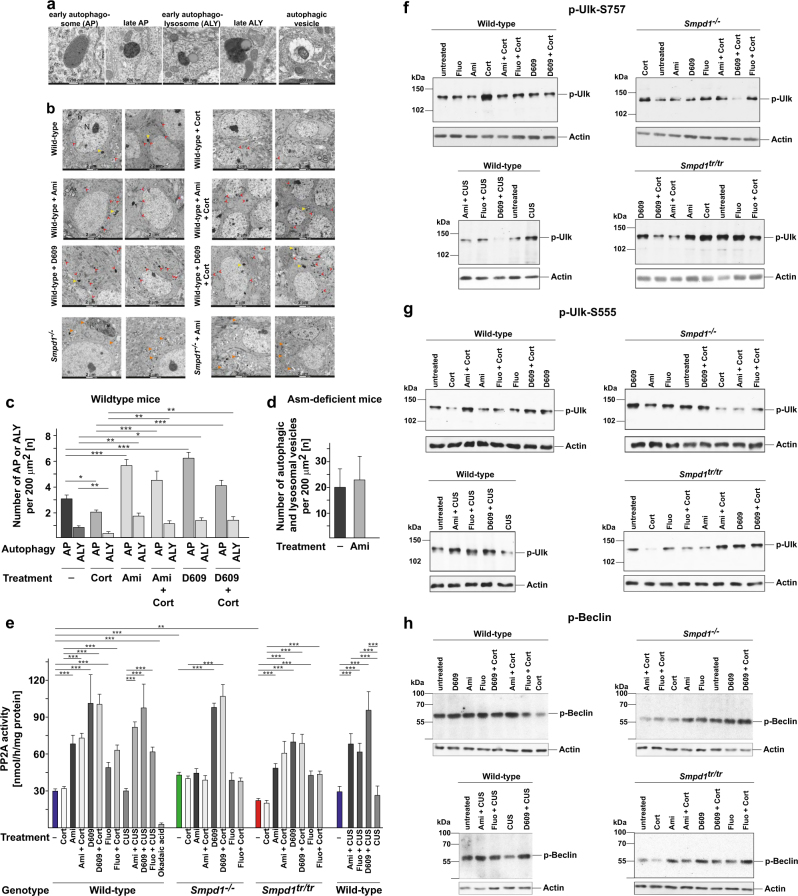

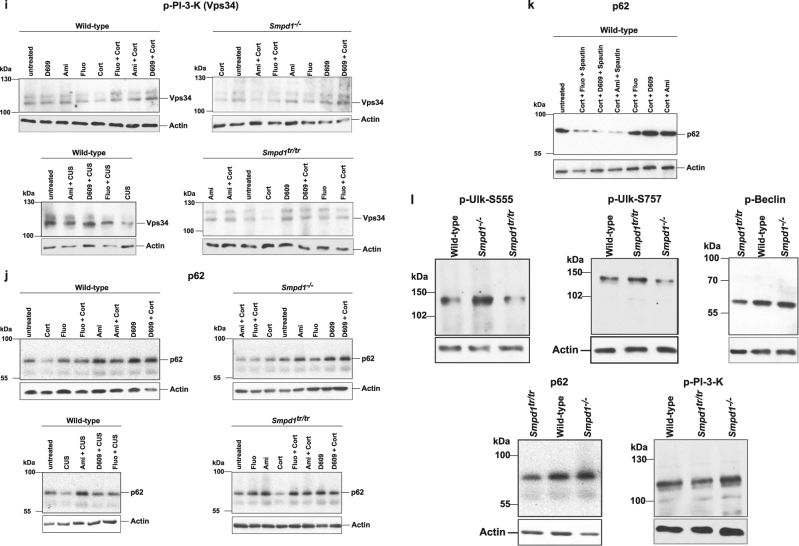
Amitriptyline and D609 induce autophagy. Wild-type (wt) and acid sphingomyelinase (Asm)-deficient mice (*Smpd1*^*−/−*^) were left untreated or were treated as indicated. The hippocampus of each mouse was isolated and subjected to analysis with transmission electron microscopy. Corticosterone (Cort) stress reduces autophagy. Amitriptyline and D609 induce autophagy in hippocampal neurons of unstressed or stressed wild-type mice. Amitriptyline has no effect in Asm-deficient cells, while D609 induces autophagy also in hippocampus neurons of these mice. Shown are typical morphologies of autophagosomal and autophagolysosomal structures (**a**) and representative transmission electron microscopy results from three independent studies (**b**). The ultrastructural features of hippocampi and the number of autophagosomes (containing morphologically intact cytosol and organelles) and autophagolysosomes (a hybrid organelle generated by the fusion of an autophagosome and a lysosome containing partially degraded cytoplasmic as well as organelle material) in untreated WT animals are in agreement with previous reports [60–62]. **c** The number of autophagosomes (AP) and autophagolysosomes (ALY) per 200 µm^2^ in hippocampal neurons of untreated or treated wildtype mice. For statistical analysis, early and late autophagosomes (red arrows) and early and late autophagolysosomes (yellow arrows) were grouped together. **d** Since Asm-deficient cells contain lipid storage bodies in the lysosomes, and cells are filled with large lysosomes that are characterized by dense concentric lamellar bodies (see also https://synapseweb.clm.utexas.edu/atlas), the lysosomes are difficult to discriminate from classical autophagosomes or autophagolysosomes. Thus, the total number of autophagic and lysosomal vesicles is given in **d**, showing that amitriptyline has no effect in Asm-deficient *Smpd1*^−/−^ mice. Examples are indicated by orange arrows in (**b**). A total area of ≥15 mm^2^ was evaluated for each condition. Shown are means ± SEM from three different animals for each condition; *p < 0.05, **p < 0.01, ***p < 0.001, ANOVA. TEM images were randomly chosen for statistical evaluation that was carried out independently by two researchers. Abbreviations are: Ap autophagosome, Aly autophagolysosome, M mitochondria, N nucleus, Ax myelin-surrounded axon. **e** Treating unstressed or stressed wild-type (wt) or acid sphingomyelinase (Asm)-transgenic mice (*Smpd1*^*tr/tr*^) with amitriptyline or fluoxetine induces the activation of phosphatase 2A (PP2A) in hippocampus extracts, whereas antidepressants have no effect on Asm-deficient mice (*Smpd1*^−/−^). D609 induces PP2A activation in all mouse strains. PP2A activity is constitutively increased in Asm-deficient mice and is decreased in Asm-transgenic mice. Stress was induced by application of corticosterone or chronic unpredictable environmental stress (CUS). PP2A activity was determined in hippocampal extracts by a colorimetric enzyme assay. Shown are the means ± SD; *n* = 5 each; **p < 0.01, ***p  <  0.001, ANOVA. **f**–**l** Treating wild-type (wt) or acid sphingomyelinase (Asm)-transgenic mice with antidepressants induces the dephosphorylation of Ulk at inhibitory serine 757 (**f**) and a concomitant phosphorylation of Ulk at activating serine 555 (**g**), the activating phosphorylation of Beclin (**h**) and PI3-K/Vps34 (**i**), and an increase in the expression of p62 (**j**). None of these effects occurs in Asm-deficient mice (**f**–**l**). Inhibiting sphingomyelin synthases with D609 resulted in the activation of Ulk, Beclin, and PI3- K/Vps34 and induced the expression of p62 in all mouse strains, regardless of Asm expression (**f**–**l**). Corticosterone or chronic unpredictable environmental stress (CUS) induced a dephosphorylation of Ulk at serine 555, induced the phosphorylation of Ulk at serine 757, reduced the phosphorylation of Beclin and Vps34, and triggered a downregulation of p62 expression in all mouse strains (**f**–**l**). The effects of stress were reversed in wild-type and Asm-transgenic mice by treatment with amitriptyline, fluoxetine, or D609. In Asm-deficient mice, only D609 reversed the effects of stress. **k** Injection of spautin-1, an inhibitor of Beclin, abrogated the effects of antidepressants and D609 on the expression of p62 in the hippocampus of stressed mice. **l** Analysis of the constitutive phosphorylation of Ulk at serine 757 and serine 555, Beclin, and PI-3-K/Vps34, and the expression of p62 in hippocampal extracts from wild-type, Asm-deficient, and Asm-transgenic mice shows increased activity of Ulk, Beclin, and PI3-K/Vps, and the expression of p62 in Asm-deficient mice compared to wild-type mice, whereas the activity and expression of these proteins is downregulated in Asm-transgenic mice. Phosphorylation or expression of Ulk, Beclin, PI3-K/Vps 34, or p62 was determined with western blots of extracts prepared from isolated hippocampi. Blots are representative for five independent experiments (for quantification please see Supplementary Figure 5)

Next, we investigated how an increase in lysosomal concentrations of sphingomyelin with a subsequent specific increase in sphingomyelin concentrations in the Golgi bodies and ceramide concentrations in the ER could be linked to MDD. We hypothesized that ceramide in the ER regulates autophagy in neurons. To test this hypothesis, we performed transmission electron microscopy studies on freshly isolated hippocampi from unstressed or stressed mice treated with amitriptyline or D609. These studies showed that amitriptyline and D609 increased the formation of autophagosomes and of autophagolysosomes in hippocampal neurons in both unstressed and corticosterone-stressed mice (Fig. 2a–c). Stress alone decreased the formation of autophagosomes and autophagolysosomes (Fig. 2b, c). Amitriptyline had no effect on autophagy in hippocampal neurons in Asm-deficient mice (Fig. 2a, d).

Collectively, the results of our EM studies indicate that antidepressants and D609 induce the formation of autophagosomes and autophagolysosomes in hippocampal neurons, a process that is abrogated in mice lacking Asm.

### ER ceramide induced by antidepressants or D609 triggers the activation of phosphatase 2A

Our EM studies raise the question of how the inhibition of Asm activity by an increase in ceramide concentrations in the ER could be linked to autophagy. Previous findings have shown that ceramide in the ER regulates the activity of PP2A [31, 32]. We therefore investigated whether amitriptyline, fluoxetine, and D609 regulate the activity of PP2A and whether this regulation is controlled by Asm expression. To this end, we treated mice with amitriptyline, fluoxetine, or D609 in the presence or absence of either corticosterone or chronic unpredictable environmental stress and measured the activity of PP2A in hippocampal extracts. The results showed that treatment with amitriptyline or fluoxetine for 12 days or treatment with D609 for only 3 days, alone or together with corticosterone or unpredictable environmental stress, results in marked activation of PP2A (Fig. 2e). In mice lacking Asm, this activation was absent after treatment with amitriptyline or fluoxetine but was induced by treatment with D609 (Fig. 2e). Mice overexpressing Asm exhibited reduced PP2A activity in hippocampal extracts before any treatment; this activity increased after treatment with amitriptyline, fluoxetine, or D609, regardless of whether the mice were stressed (Fig. 2e).

Controls showed that protein levels of PP2A did not change after any of the treatments (Supplementary Fig. 5a). PP2A activity was abrogated by 3 days of intraperitoneal treatment of mice with okadaic acid, an inhibitor of PP2A, thereby proving the specificity of the enzyme measurements.

### Antidepressants and D609 induce autophagy

PP2A has previously been linked to cellular autophagy [33], and autophagy has previously been linked to MDD [34, 35]. We therefore hypothesized that antidepressants may increase cellular autophagy via alterations in sphingomyelin–ceramide concentrations and may thereby act against MDD. A key regulator of autophagy is the kinase Ulk [36, 37]. Ulk is activated by phosphorylation of serine 555 and by dephosphorylation of serine 757 [38, 39]. Thus, Ulk may be a target of PP2A, a phosphatase. Western blot studies of hippocampal extracts from mice exposed to corticosterone stress or chronic unpredictable environmental stress found hyper-phosphorylation of Ulk at the inactivating serine 757 and dephosphorylation of Ulk at the activating serine 555, compared to the signal in extracts from untreated mice (Fig. 2f, g). Treating stressed mice with amitriptyline or fluoxetine for 12 days or with D609 for 3 days mediated the phosphorylation of Ulk at serine 555 and its dephosphorylation at serine 757, a finding corresponding to activation of the kinase (Fig. 2f, g). In Asm-deficient mice, corticosterone or chronic unpredictable environmental stress also induced hyperphosphorylation of serine 757 in Ulk, similar to that observed in wt mice, but Asm-deficient mice did not respond to amitriptyline and fluoxetine with dephosphorylation of Ulk at serine 757 or phosphorylation of Ulk at serine 555, a finding consistent with the notion that amitriptyline and fluoxetine target Asm (Fig. 2f, g). In contrast, unstressed or stressed Asm-deficient mice responded rapidly to D609 with dephosphorylation of Ulk at serine 757 and phosphorylation of Ulk at serine 555 (Fig. 2f, g). Stress resulted in strong hyperphosphorylation of Ulk at serine 757 and in hypophosphorylation of Ulk at serine 555 in the hippocampus of Asm-transgenic mice; these changes were corrected by treatment with amitriptyline, fluoxetine, or D609 (Fig. 2f, g).

Ulk phosphorylates Beclin, a key regulator of autophagy, which is activated by phosphorylation [40]. A downstream target of activated Beclin is Vps34, a phosphatidylinositol 3-kinase (PI3-kinase) type III [40]. We therefore measured the phosphorylation of Beclin and Vps34 in unstressed or stressed wt, Asm-deficient, and Asm-transgenic mice. The results are very similar to those obtained with Ulk and show a dephosphorylation (i.e., inactivation) of Beclin and Vps34 in all three genotypes of mice after corticosterone stress or unpredictable environmentally mediated stress. Amitriptyline or fluoxetine induced phosphorylation and thereby activation of Beclin in unstressed or stressed wt and Asm-transgenic mice but not in Asm-deficient mice (Fig. 2h, i). D609 rapidly induced phosphorylation of Beclin in all three genotypes of mice (Fig. 2h, i).

Controls showed that stress, amitriptyline, fluoxetine, D609, or a combination of these treatments did not change the protein expression of Ulk, Beclin, and PI3-K/Vps34 in the hippocampus (Supplementary Fig. 5b-d).

**Fig. 3:**
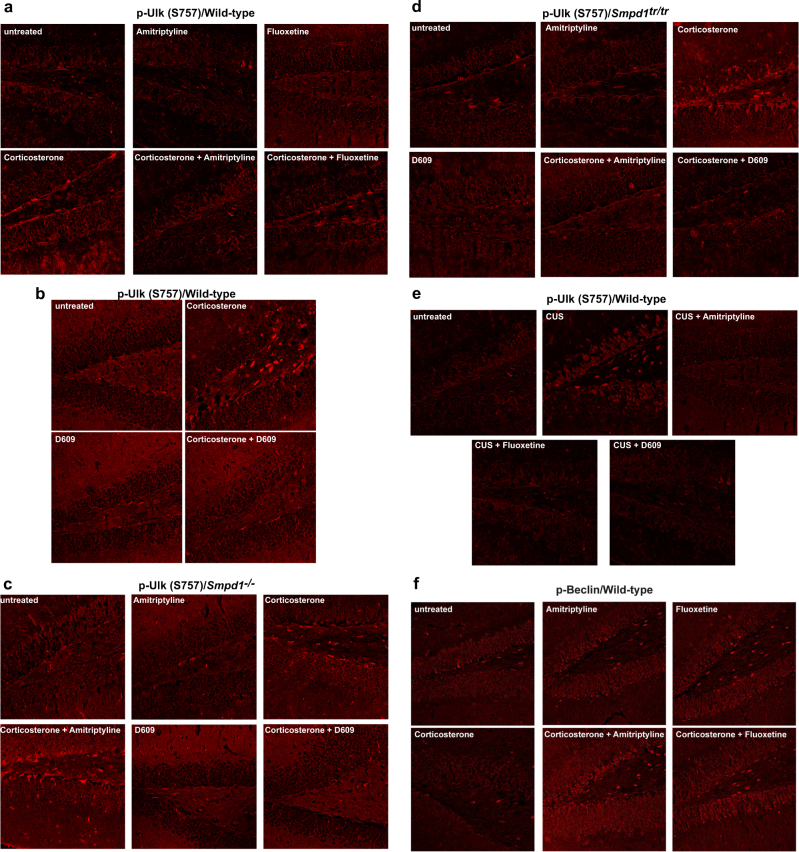

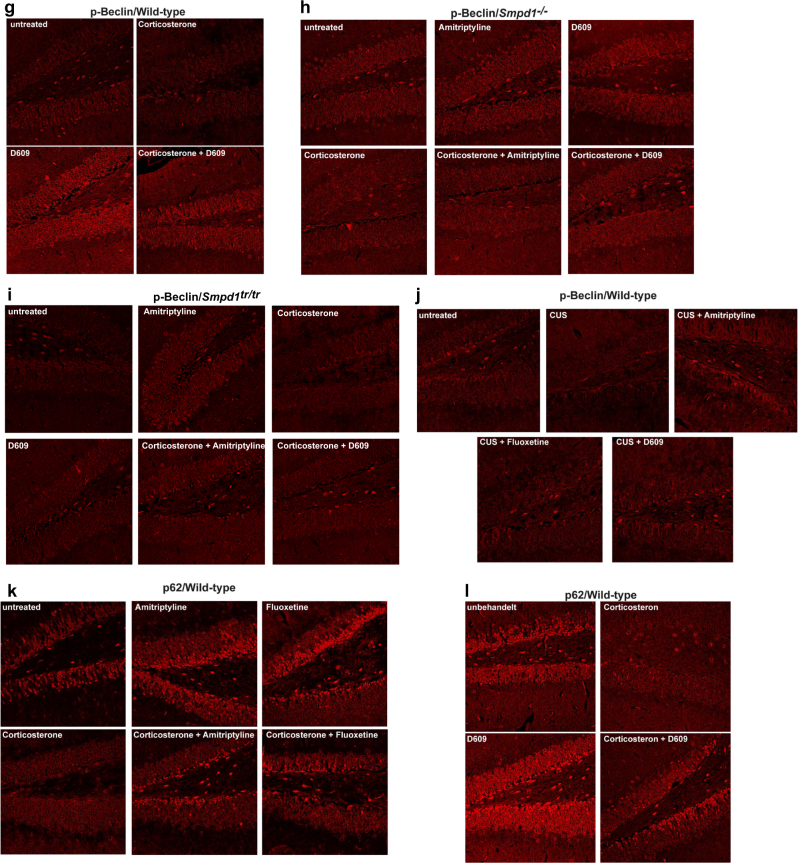

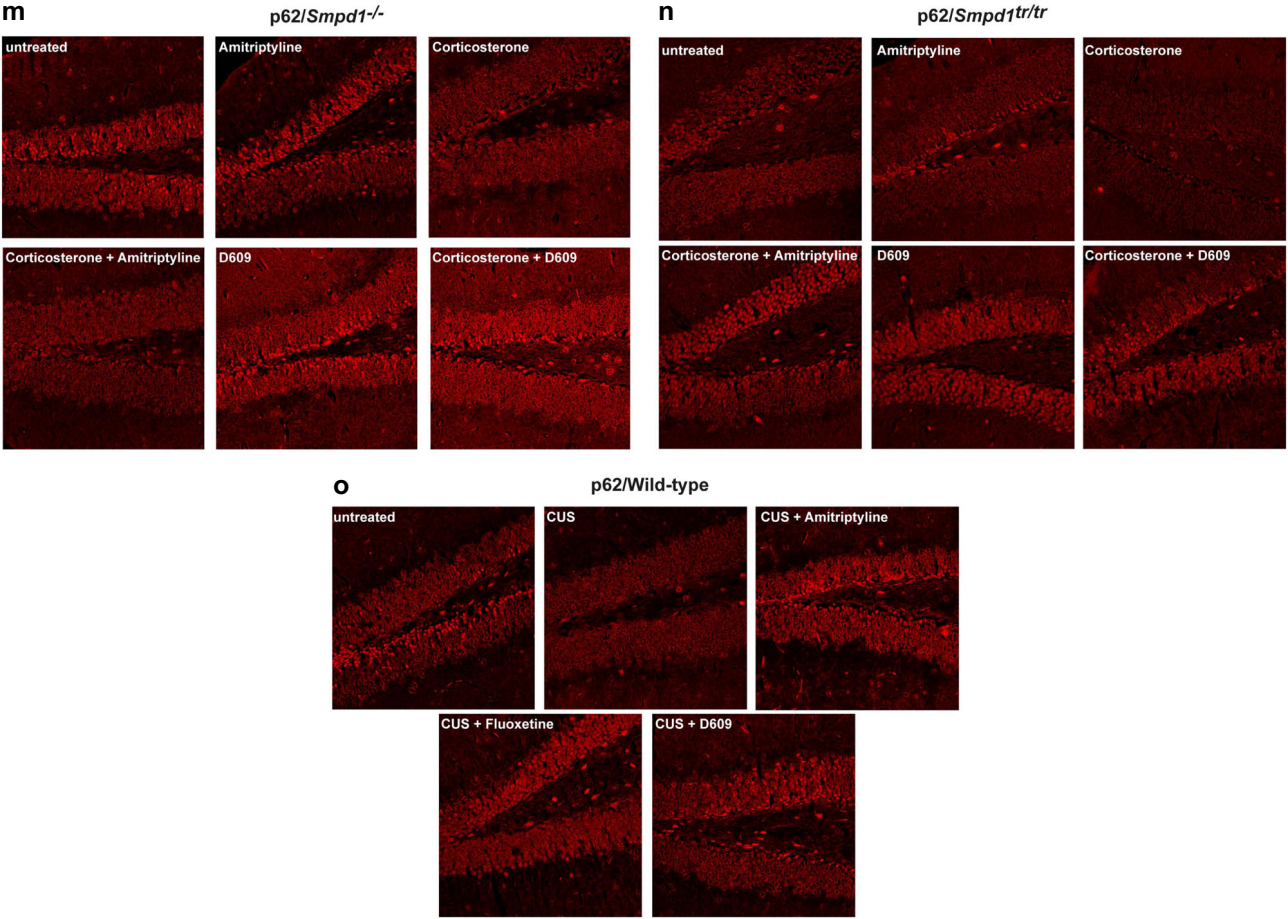
(a-o) Antidepressants and D609 induce activation of Ulk and Beclin as well as expression of p62 in the Cornu ammonis of the hippocampus. Phosphorylation or expression of Ulk, Beclin, or p62 was determined by immunostaining paraffin sections from the hippocampus of wild-type, Asm-deficient, and Asm-transgenic mice with phosphospecific antibodies to Ulk serine 757 and phospho-Beclin or with anti-p62 antibodies, followed by staining with Cy3-labeled secondary antibodies. Mice were stressed by corticosterone or chronic unpredictable environmental stress (CUS) and treated with amitriptyline, fluoxetine, or D609. Samples were analyzed by confocal microscopy. Shown are representative results from 6 mice each. For quantification please see Supplementary Figure 6

Next, we investigated the expression of p62, a protein that is crucial for the formation of autophagosomes [41], in the hippocampus after treatment with antidepressants or D609. Corticosterone or chronic unpredictable stress reduced the expression of p62 in the hippocampus of wt, Asm-deficient, and Asm-transgenic mice (Fig. 3f).

Treatment with amitriptyline or fluoxetine increased p62 expression in the hippocampus of unstressed or stressed wt and Asm-transgenic mice but had no effect on Asm-deficient mice (Fig. 2j), whereas D609 increased hippocampal p62 expression in all three mouse strains (Fig. 2j). Spautin-1, an inhibitor of Beclin, prevented the increase of p62 expression in wt mice treated with amitriptyline, fluoxetine, or D609 (Fig. 2k).

Direct comparison of phospo-Ulk, phospho-Beclin, phospho-PI3-K/Vps34, and p62 levels in hippocampal extracts showed increased constitutive autophagy in Asm-deficient mice and decreased autophagy in Asm-transgenic mice compared to wt mice (Fig. 2l).

All western blot studies were controlled for total protein and by actin western blots. Normalization to actin was used to quantify all blots (Supplementary Fig. 6a-f).

The protein mammalian target of rapamycin (mTOR) has been shown to be a very important regulator of autophagy [42]. However, our studies did not show any effect of amitriptyline, fluoxetine, or D609 on mTOR phosphorylation or expression (Supplementary Fig. 7a). In addition, phosphorylation of mTOR did not differ between wt, Asm-deficient, and Asm-transgenic mice (Supplementary Fig. 7a).

Collectively, the western blot studies demonstrate the activation of autophagy proteins in the hippocampus upon treatment with antidepressants or D609. A deficiency in Asm activity results in a constitutive increase in autophagy; it also abrogates the effects of amitriptyline but not those of D609. Overexpression of Asm constitutively reduces autophagy in the hippocampus.

### Immunofluorescence studies confirm an Asm-dependent induction of autophagy in the hippocampus upon treatment with antidepressants or D609

To confirm the results of the western blot studies and to unambiguously localize the induction of autophagy to the hippocampus upon inhibition of Asm activity by antidepressants or upon treatment with D609, we performed immunofluorescence studies of the hippocampus. These results were consistent with those from the western blot analyses: corticosterone or chronic unpredictable environmental stress increased the phosphorylation of Ulk at serine 757 (Fig. 3a–e), induced the dephosphorylation of Beclin (Fig. 3f–j), and mediated a decrease in the expression of p62 (Fig. 3k–o) in wt mice. The change in the phosphorylation status of Ulk and Beclin or the expression of p62 occurred in the neurogenic subgranular zone of the hippocampus but was also present in other cells. Treatment with amitriptyline, fluoxetine, or D609 reversed the effects of stress on the phosphorylation of Ulk at serine 757 (Fig. 3a, b, e) and of Beclin (Fig. 3f, g, j) and restored the expression of p62 (Fig. 3k, l, o) in wt mice. Asm-deficient mice showed a phosphorylation of Ulk at serine 757, a dephosphorylation of Beclin, and a reduction in p62 expression after several forms of stress, but these mice did not respond to amitriptyline (Fig. 3c, h, m). In contrast, all stress-induced changes in the hippocampus of these mice were reversed by treatment with D609 (Fig. 3c, h, m). Compared to wt control mice, mice overexpressing Asm exhibited a constitutive increase in the phosphorylation of Ulk at serine 757 and a decrease in the phosphorylation of Beclin and in the expression of p62 (Fig. 3d, i, n). These changes became even more severe with corticosterone administration or chronic unpredictable stress and were corrected by amitriptyline or D609 (Fig. 3d, i, n). Anti-Ulk or anti-Beclin staining of hippocampus sections did not reveal any differences in protein expression (not shown). The fluorescence levels in 20 cells per section at the neurogenetic zone of the dentate gyrus (in total, 120 cells per group) were also quantified (Supplementary Fig. 8). In addition, we controlled for similar thickness of the sections by staining with Cy3-coupled anti-actin antibodies and analysis of the fluorescence signal in 20 cells per section using conventional fluorescence microscopy (Supplementary Fig. 9).

The results of these studies confirm the induction of the autophagic cascade in the hippocampus by antidepressants and D609. They also show that Asm expression determines the effects of amitriptyline.

### Antidepressants and D609 induce autophagy and the formation of autophagolysosomes via endoplasmic ceramide, PP2A activation, Ulk, Beclin, p62, and Lc3B

**Fig. 4:**
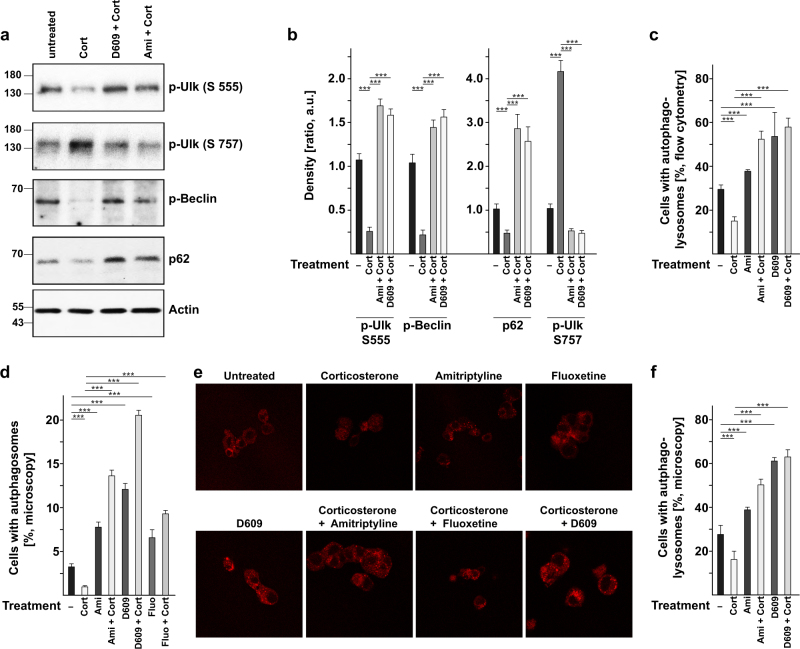
Amitriptyline or D609 activates autophagy in corticosterone-stressed PC-12 cells. **a**, **b** Stress increases the inhibitory phosphorylation of Ulk (S 757) and reduces the activating phosphorylation of Ulk (S 555) and Beclin and also reduces the expression of p62. These effects are reversed by treatment with amitriptyline or D609. Shown are representative western blots (**a**) and the quantitative analysis (**b**) from PC-12 extracts. The western blots were quantified with Image J software; ***p < 0.001, ANOVA. **c** To directly show the impact of amitriptyline and D609 on the fusion of autophagosomes with lysosomes, we transfected PC-12 cells with a RFP–GFP–Lc3B tandem construct, stressed them with corticosterone or left the cells unstressed, and simultaneously treated them with amitriptyline or D609. Phagolysosomal fusion was determined by flow cytometry measurements of the proportion of GFP-low/RFP-high vs GFP-high/RFP high cells, because the low pH of lysosomes quenches the GFP signal. Shown is the quantitative analysis of cells with autophagosomes from six independent studies; mean ± SD; ***p < 0.001, ANOVA. **d–f** To demonstrate the formation of autophagosomes after treatment of stressed or unstressed PC-12 cells with amitriptyline or D609, we transfected PC-12 cells with a RFP-p62 (**d**, **e**) or a RFP–Lc3B (**f**) construct. The formation of autophagosomes is indicated by large punctae in the cells. Cells were analyzed by fluorescence microscopy and these punctae were counted in 25 cells per sample and in six independent experiments (total of 150 cells) (**d**, **f**). A representative result is shown in **e**. Given is the mean ± SD of the number of dots positive for p62 or Lc3B per cell; ***p < 0.001, ANOVA

To confirm the induction of the autophagic cascade and to directly show an increase in the formation of autophagosomes and autophagolysosomes after treatment with amitriptyline or D609, we used cultured PC-12 cells. First, we investigated whether the in vivo effects of antidepressants and D609 on autophagy proteins can be recapitulated in cultured PC-12 cells. The results showed a decrease in the activating phosphorylation of Ulk at serine 555, an increase in the inhibitory phosphorylation of Ulk at serine 757, and reduced phosphorylation of Beclin and Vps 34, as well as decreased expression of p62 upon application of corticosterone (Fig. 4a, b). Concomitant treatment with amitriptyline or D609 reversed these effects (Fig. 4a, b).

To demonstrate a role of PP2A in the effects of amitriptyline and D609, we treated stressed or non-stressed PC-12 cells with amitriptyline or D609 in the presence or absence of okadaic acid, an inhibitor of PP2A. Treatment with okadaic acid abrogated Ulk and Beclin activation by amitriptyline or D609, as well as induction of autophagy, a finding indicating the significance of PP2A for the effects of amitriptyline and D609 (Supplementary Fig. 10a).

Next, to investigate whether ceramide in the ER or the plasma membrane induces PP2A activation, we performed detailed confocal microscopy studies on PC-12 cells treated with amitriptyline or D609 with or without corticosterone. Cells were co-stained with Cy3-coupled anti-ceramide and fluorescein isothiocyanate (FITC)-labeled anti-Lamp1 (a lysosomal marker), FITC-anti-calreticulin (an ER marker), or FITC-anti-β1-integrin (a plasma membrane marker) antibodies. The studies showed an intracellular accumulation of ceramide in the ER after amitriptyline or D609 and a reduction in lysosomal ceramide concentrations after amitriptyline, but no change in ceramide concentrations in the plasma membrane (Supplementary Fig. 10b). Finally, we investigated whether sphingomyelin activates PP2A and determined that it does not (Supplementary Fig. 10c).

To directly show that antidepressants and D609 regulate autophagy, we co-transfected PC-12 cells with a red fluorescent protein–green fluorescent protein–light chain 3 β (RFP–GFP–Lc3B) tandem construct and determined the fusion of autophagosomes with lysosomes. The fusion of autophagosomes with acidic lysosomes reduces the pH-sensitive fluorescence of GFP, whereas the fluorescence of RFP is not affected by the pH. The samples were then analyzed by flow cytometry. These studies showed that corticosterone treatment reduces autophagy. Amitriptyline and D609 triggered autophagy, in particular in stressed cells (Fig. 4c). In addition, we transfected PC-12 cells with an RFP-p62 (Fig. 4d, e) or an RFP–Lc3B (Fig. 4f) expression construct and used microscopy analysis to determine RFP-positive punctae that indicate the formation of autophagosomes. These studies showed that amitriptyline or D609 induces the formation of autophagosomes and reverses the negative impact of stress on autophagosome formation (Fig.  4d–f).

Collectively, these studies show that intracellular accumulation of ceramide, most likely in the ER but possibly also in the inner leaflet of the plasma membrane, mediates the activation of PP2A, Ulk, and Beclin and finally induces autophagy upon treatment of cells with amitriptyline or D609.

### Inhibition of autophagy abrogates the effects of antidepressants and D609 on neuronal proliferation and maturation, as well as on behavior

To determine whether the cascade from increased concentrations of sphingomyelin in lysosomes and Golgi bodies and increased concentrations of ceramide in the ER to increased PP2A activity, the activation of Ulk, the phosphorylation and activation of Beclin, and thereby the stimulation of autophagy acts against MDD, we examined neurogenesis and behavior in unstressed and stressed mice treated with antidepressants or D609 in the presence or absence of spautin-1, an inhibitor of Beclin. We hypothesized that this inhibitor would prevent the effects of antidepressants or D609.

**Fig. 5:**
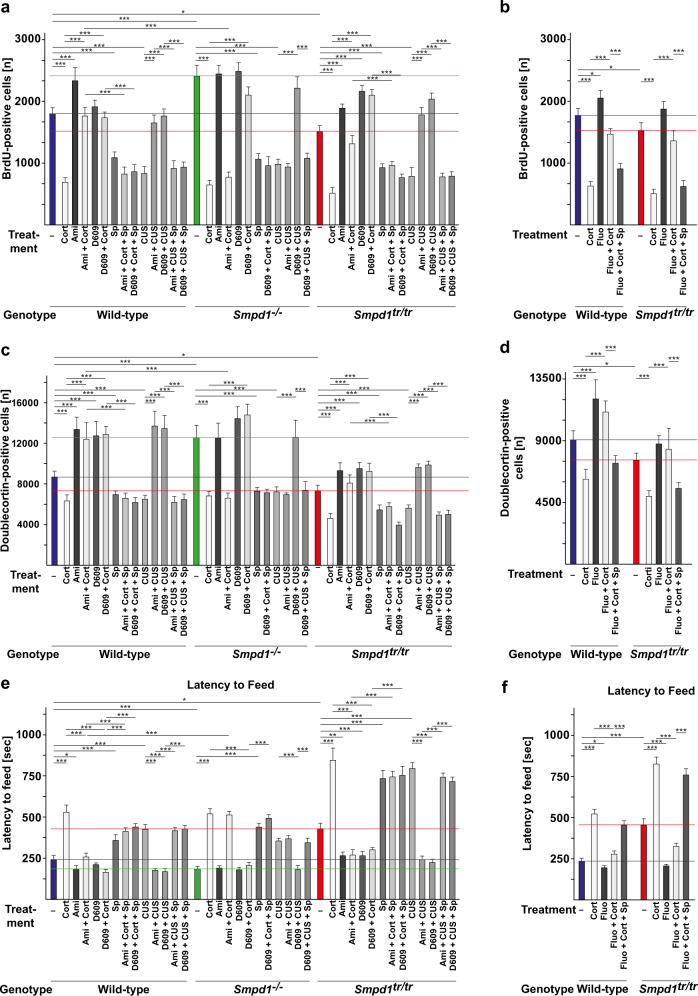

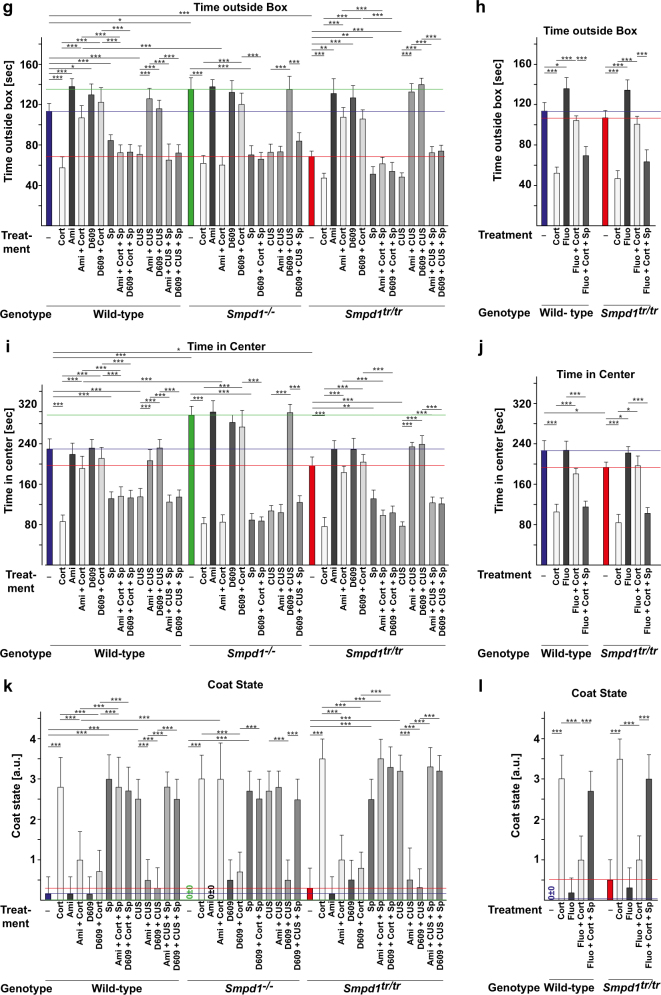

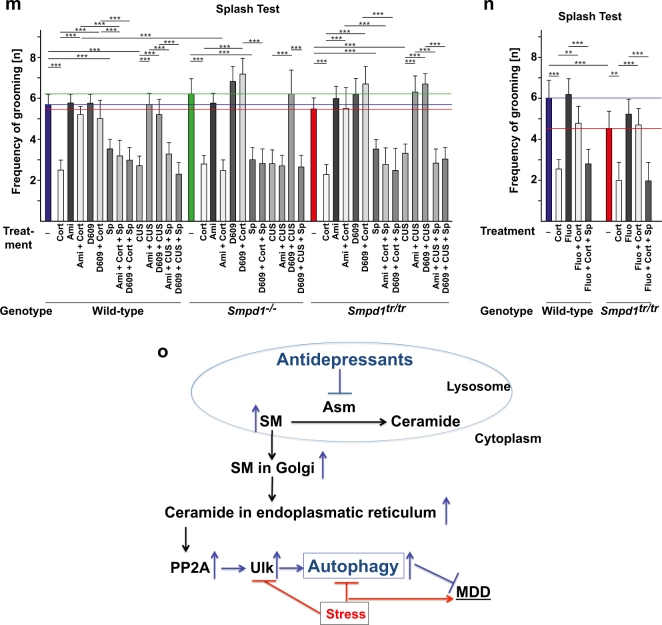
Antidepressants and D609 regulate neurogenesis, neuronal maturation, and neuronal behavior via autophagy. **a**–**n** Stress reduces neurogenesis (**a**, **b**) and neuronal maturation (**c**, **d**) and induces symptoms typical of major depressive disorder (**e**–**n**). Amitriptyline (Ami) and fluoxetine (Fluo) normalize all of these effects in wild-type (wt) and acid sphingomyelinase (Asm)-transgenic mice (*Smpd1*^*tr/tr*^), but have no effect on Asm-deficient mice (*Smpd1*^−*/−*^) (**a**–**n**). In contrast, D609 normalizes neurogenesis (**a**, **b**), neuronal maturation (**c**, **d**), and depression-like behavior, as determined by the novelty-suppressed feeding test (**e, f**), the light/dark box test (**g, h**), the open-field test (**i**, **j**), the coat state test (**k**, **l**), and the splash test (**m**, **n**) in all three mouse strains after stress, regardless of Asm expression. The effects of amitriptyline, fluoxetine, and D609 are abrogated by pharmacological inhibition of Beclin with spautin-1 (Sp) (**a**–**n**). The means ± SD of the quantitative analysis of serial sections of BrdU or doublecortin stainings of the hippocampus are shown. The studies were performed with six mice per group; mean ± SD; *p < 0.05, **p < 0.01, ***p < 0.001, ANOVA. The colored lines indicate the untreated levels in wt (blue), *Smpd1*^−*/−*^(green) and *Smpd1*^*tr/tr*^ (red) mice. **o** Antidepressants upregulate sphingomyelin (SM) in lysosomes and in the Golgi, and finally ceramide in the endoplasmic reticulum (ER), by treatment with antidepressants. D609 induces a rapid and direct upregulation of ceramide in the ER. Ceramide in the ER activates PP2A, which dephosphorylates Ulk at serine 757, thereby allowing activity of the kinase and subsequent phosphorylation of Beclin and PI-3-K/Vps34, upregulation of the expression and function of p62 and Lc3B, and thereby the formation of autophagosomes that fuse with lysosomes to form autophagolysosomes. Inhibition of this pathway by spautin-1, an inhibitor of Beclin, abrogates the effects of antidepressants and D609 on neurogenesis, neuromaturation, and neurobehavior and induces depressed behavior by itself

The results showed that corticosterone stress or chronic unpredictable environmental stress decreased neurogenesis, as determined by bromodeoxyuridine (BrdU) incorporation (Fig. 5a, b); decreased neuronal maturation (Fig. 5c, d), as measured by doublecortin staining; and induced depressive behavior in wt, Asm-deficient, and Asm-transgenic mice (Fig. 5e–n), a finding consistent with previous results [22]. Asm-deficient mice exhibited a constitutive increase in neurogenesis and neuronal maturation (Fig. 5a–d) and a decrease in depressive behavior (Fig. 5e–n). Asm-transgenic mice exhibited a constitutive decrease in neurogenesis and neuronal maturation (Fig. 5a–d) and also exhibited depressive behavior, even without stress (Fig. 5e–n). Treatment with amitriptyline or fluoxetine improved neurogenesis and neuronal maturation and normalized behavior in stressed wt and Asm-transgenic mice, whereas this treatment had no effect on Asm-deficient mice (Fig. 5a–n). D609 rescued all mouse genotypes, regardless of Asm expression, from the effects of stress (Fig. 5a–n). The beneficial effects of amitriptyline, fluoxetine, and D609 on neurogenesis and behavior were abrogated by treatment with spautin-1, which affected neurogenesis, neuronal maturation, and behavior to a similar degree as did corticosterone or unpredictable environmental stress (Fig. 5a–n).

Because the delayed action of classic antidepressants such as amitriptyline or fluoxetine is an important clinical problem, we performed a time-course analysis of the effects of D609 on behavior. The results show that D609 improves behavior after only 36 h of treatment, although a complete reversal of stressed behavior requires 3 days of treatment with D609 (Supplementary Fig. 11).

## Discussion

The results of the present studies indicate that autophagy is a signaling pathway that is crucially involved in the regulation of MDD and the effects of antidepressants (Fig. 5o). Exogenous environmental stress or corticosterone inhibits autophagy, whereas treatment with antidepressants, such as amitriptyline and fluoxetine, activates autophagy. On the basis of this information, we found that D609, which blocks sphingomyelin synthase 1 and 2 ^28, 29^ and thereby directly induces an increase in the concentration of ceramide in the ER, triggers autophagy and functions as a novel antidepressant acting within 36–72 h. Such fast-acting antidepressants would be very important clinically, because the long delay associated with the onset of the clinical activity of antidepressants is one of the main problems in the treatment of patients with MDD.

D609, its derivatives, or other drugs that directly target sphingomyelin synthases or autophagy may form a novel class of fast-acting antidepressants. D609 has also been shown to induce ceramide synthesis, which would further increase the concentration of ceramide in the ER and promote the antidepressive effect of D609 [43].

Our findings are consistent with important clinical observations:

First, tricyclic antidepressants show a lag time of 2–4 weeks before they begin to demonstrate a clinical effect in patients with MDD. This delay is consistent with the slow accumulation of sphingomyelin and, in particular, ER ceramide concentrations in mice treated with antidepressants. Even in Asm-deficient mice lysosomal storage disease with accumulation of sphingomyelin develops slowly [23, 34], a finding indicating that, at least for some time, compensatory mechanisms can balance Asm deficiency. These compensatory mechanisms require definition but could include any protein involved in ceramide and sphingomyelin synthesis or in the transport of sphingomyelin from the Golgi bodies to the lysosomes.

Second, stimuli that promote autophagy act against MDD. For instance, lithium is often used to treat patients with MDD and, in particular, to amplify the effects of antidepressants. This treatment is consistent with the notion that lithium induces and facilitates autophagy [44]. Electroconvulsive therapy has been shown to trigger autophagy in the hippocampus [45], and the rapid onset but transient nature of autophagy after electroconvulsive therapy may explain the rapid and transient effect of this treatment. The antidepressant effects of physical activity may also be explained via increased autophagy [46, 47]. Our studies are also consistent with previous studies showing that tricyclic antidepressants induce autophagy [34]. However, these studies did not identify any targets or signaling pathways induced by antidepressants to trigger autophagy.

Third, MDD not only causes central nervous system (CNS) symptoms but is also associated with many somatic symptoms, including cardiovascular diseases, inflammation, and osteoporosis [2–4, 48]. These symptoms cannot be explained by altered neurogenesis in the hippocampus, but they may be explained by a change in autophagy in non-neuronal cells.

Fourth, many patients with MDD exhibit a circadian rhythm of symptoms, with a high (weaker symptoms) in the afternoon or evening and more severe symptoms in the morning. This finding is consistent with the circadian rhythm of autophagy in cells, with a peak around 1:00 p.m. and a nadir around 1:00 a.m. [49]. Such a rhythm, again, is difficult to explain with a hypothesis of neurogenesis alone.

We have previously shown that Asm-transgenic mice exhibit constitutive behavior corresponding to that seen with mild depression [22, 30], a finding consistent with our present findings. However, we have also reported that an increase in lysosomal concentrations of ceramide in mice heterozygous for acid ceramidase induces MDD [22]. These findings suggest that the reduction in lysosomal ceramide concentrations that is induced by tricyclic antidepressants also contributes to the antidepressive effects of these drugs, at least under circumstances of increased lysosomal ceramide concentrations.

We therefore suggest the following model to explain the dual role of lysosomal ceramide versus ER ceramide: an increase of ceramide in the ER induces autophagy as shown here. A decrease of ceramide in lysosomal membranes alters their biophysical properties and promotes fusion of lysosomes with autophagosomes, the process of lysosomal reformation from autophagolysosomes, or both [50, 51].

Lysosomal reformation requires an outward budding of new lysosomes from autophagolysosomes [50, 51]. Because ceramide in the inner leaflet of the lysosomal membrane promotes an inward budding, this process might be inhibited by an increased intralysosomal concentration of ceramide that would finally reduce and inhibit autophagy, because newly formed autophagosomes do not find enough lysosomes with which they can fuse. In this scenario, the reduction of lysosomal ceramide concentrations and the increase of ceramide concentrations in the ER, both of which are induced by antidepressants, may act in concert to induce autophagy (via increased ER ceramide concentrations) and promote autophagosome–lysosome fusion and lysosomal reformation (via decreased lysosomal ceramide concentrations).

In addition, lysosomes containing increased amounts of ceramide may fuse with the plasma membrane, resulting in ceramide-enriched membrane platforms at the cell surface [52, 53]. These membrane platforms have previously been shown to trap and cluster receptors and seem to be particularly involved in many stress signals. Thus, these platforms may trigger stress by the reorganization and selective activation of stress receptors. Inactivation of these pathways by reduction of lysosomal ceramide levels may act against stress. Thus, although the multiple roles of ceramide in MDD require further definition, several recent patient and genetic studies principally confirm our data that ceramide plays an important role in the pathophysiology of MDD [54–56].

At present it is unknown how stress may influence autophagy. We investigated two forms of stress, environmental stress and corticosterone, and detected a reduction of autophagy in the hippocampus and in cultured cells after stress. Various forms of stress may thus converge to inhibit autophagy or to increase the need for autophagy by inducing premature aging and dysfunction of organelles and proteins. If the neurons in the hippocampus and in somatic cells cannot respond to this increased requirement of autophagy, dysfunctional proteins and organelles accumulate and may cause the symptoms of MDD.

As outlined above, neither the monoamine hypothesis [8] nor a reduction of neurogenesis in the hippocampus [11–14] fully explains the pathogenesis of MDD and the molecular effects of antidepressants. The present study does not exclude neurotransmitter uptake or neurogenesis as targets in the treatment of MDD, but it indicates that additional factors are necessary for overcoming MDD. Thus, antidepressants may induce a dynamic turnover of synapses [57] and thereby promote the integration of novel neurons into neuronal networks that may be impaired by stress. In addition, recent publications demonstrated a link between the extracellular matrix in the hippocampus and MDD [58]. The regulation of the turnover of membrane transporters that control neurotransmitter uptake, of synapses, and of the extracellular matrix may require fully active autophagosome formation and fusion of autophagosomes with lysosomes. Thus, a reduction of autophagy by stress may affect many cell functions and thereby finally result in MDD.

In summary, our studies identified a novel mechanism by which antidepressants act to treat MDD (Fig. 5o). We also identified D609 as a fast-acting antidepressant that may lead to the development of a new class of antidepressants with a rapid onset of action.

## Methods

### Mice and treatments

Asm-deficient mice (*Smpd1*^*−*^^*/−*^) were originally obtained from Dr. R. Kolesnick (Memorial Sloan-Kettering Cancer Center, New York, NY, USA). The mice were backcrossed for more than ten generations on the C57BL/6 Harlan background. The mice show an age-dependent overall accumulation of sphingomyelin at 10–12 weeks of age and develop Niemann–Pick syndrome A or B at more than 16 weeks of age. Thus, we used mice aged 6–9 weeks [23, 24].

In Asm-transgenic mice (*Smpd1*^*tr/tr*^), the murine *Smpd1* cDNA was expressed under the control of the ubiquitous cytomegalovirus (CMV) immediate-early enhancer/chicken β-actin promoter fusion promoter [22]. A loxP-flanked STOP cassette was included between the promoter and the transgene to allow conditional expression by crossing with E2A-Cre mice [22] and expression of a Cre recombinase.

Amitriptyline (120 mg/L; Sigma, Deisenhofen, Germany) or fluoxetine (120 mg/L; Ratiopharm, Ulm, Germany) was administered to mice for 5 or 12 days via their drinking water. Drugs were dissolved in water, which was changed every 48 h. D609 (500 mg/L; Tocris, Bristol, UK) was also administered via the drinking water for 3 days, and the water was replaced every 4–6 h. Corticosterone (Sigma) was administered via the drinking water at 0.25 mg/mL for 15 days. If amitriptyline, fluoxetine, D609, and corticosterone were administered together, the corticosterone treatment was initiated and was followed by the application of amitriptyline or fluoxetine for 5 or 12 days, or of D609 for 3 days. BrdU was injected four times (every 2 h) at a dose of 75 mg/kg 1 day before the mice were sacrificed. Spautin-1 was injected intraperitoneally at 30 mg/kg once daily for 3 days before the experiments. The time course of treatments is given in Supplementary Fig. 10.

In the chronic unpredictable stress model, the mice were challenged with unpredictable environmental stress for 3 weeks, i.e., a shift of the day-light cycle (for light/dark successions of 30 min every 24 h once each week), reversal of the light/dark cycle once each week, 3 h of 45° tilting of the cage twice each week, water deprivation for 14 h once each week, and predator sounds (15 min) three times each week. Amitriptyline or fluoxetine administration was initiated 9 days after the stress was started and was applied for the next 12 days. D609 administration was initiated after 18 days and was applied for the next 3 days. We used male and female mice. All studies were performed in accordance with animal permissions of the Regierungspraesidium Düsseldorf and the Institutional Animal Care and Use Committee (IACUC) of the University of Cincinnati (Cincinnati, OH, USA).

### HPLC-MS/MS analysis of ceramides and sphingomyelins

Hippocampal tissue samples were homogenized in aqueous buffered solution on ice with a Bead Ruptor 12 (Omni International, Kennesaw, GA, USA). Aliquots of the homogenates (20 μL, which corresponded to tissue equivalents of 1 mg) were subjected to lipid extraction with 1.5 mL methanol/chloroform (2:1, v:v). The extraction solvent contained C_17_-ceramide and C_16_-d_31_-sphingomyelin (both Avanti Polar Lipids, Alabaster, AL, USA) as internal standards. Extraction was facilitated by incubation at 48 °C with gentle shaking (120 rpm) overnight. Pellets of purified cell organelles (lysosomes, Golgi bodies, and ER) were loosened by ultrasonication on ice for 15 min, followed by lipid extraction as described for hippocampal tissue. After lipid extraction, samples were saponified with 150 μL 1 M methanolic KOH for 2 h at 37 °C with gentle shaking (120 rpm). Samples were then neutralized with 12 μL glacial acetic acid and centrifuged at 2200 × *g* for 10 min at 4 °C. Organic supernatants were evaporated to dryness through vacuum centrifugation with a Savant SpeedVac concentrator (Thermo Fisher Scientific, Dreieich, Germany). Dried residues were reconstituted in 200 μL of a 95:5 (v:v) mixture of HPLC eluents B:A (see below), thoroughly vortexed for 10 min at 1500 rpm, centrifuged at 2200 × *g* for 10 min at 4 °C, and subjected to mass spectrometric sphingolipid quantification. All analyses were conducted with a 1200 series high-performance liquid chromatograph (HPLC) coupled to a quadrupole time-of-flight (QTOF) 6530 mass spectrometer (Agilent Technologies, Waldbronn, Germany) operating in the positive electrospray ionization (ESI+) mode.

Chromatographic separations were achieved on a ZORBAX Eclipse Plus C8 column (2.1 × 150 mm, 3.5 µm; Agilent Technologies) at 30 °C. The injection volume per sample was 10 μL. A mobile phase system consisting of water (eluent A) and acetonitrile/methanol (1:1, v:v; eluent B), both acidified with 0.1% formic acid, was used for gradient elution at an initial composition of 10:90 (A:B, v:v) and a flow rate of 0.7 mL/min. The total run time for one analysis, including re-equilibration of the HPLC system, was 34 min. For mass spectrometric measurements, the following ion source settings were adjusted: sheath gas temperature, 380 °C; sheath gas flow, 12 L/min of nitrogen; nebulizer pressure, 45 psig; drying gas temperature, 360 °C; drying gas flow, 10 L/min of nitrogen; capillary voltage, 4500 V; fragmentor voltage, 155 V; and nozzle voltage, 2000 V. Ceramides and sphingomyelins, both eluting at various retention times depending on their chain length, were analyzed in tandem mass spectrometry (MS/MS) mode using the fragmentation of the precursor ions into the product ion *m/z* 264.270 (for ceramides) or *m/z* 184.074 (for sphingomyelins) [59]. A collision energy of 25 eV was applied for collision-induced dissociation (CID) of all sphingolipid species investigated. Quantification was performed by means of external calibration with the MassHunter software (Agilent Technologies). Calibration curves of reference ceramides and sphingomyelins were performed from 1 to 100 pmol per injection and were constructed by linear fitting using the least-squares linear regression calculation. The resulting slope of the calibration curve was used to calculate the concentration of the respective analyte in the samples. Determined sphingolipid amounts were normalized to the actual protein content of the homogenate or cell organelle pellet used for extraction.

### Measurement of ceramide by the diacylglycerol kinase method

Mice were sacrificed, the hippocampus was removed and shock frozen; 200 μL H_2_O was added to the frozen sample, and the sample was immediately homogenized with a tip sonicator. Aliquots were removed to determine protein concentrations, and the remaining samples were extracted in CHCl_3_:CH_3_OH:1 N HCl (100:100:1, v/v/v); the lower phase was collected, dried, resuspended in 20 μL of a detergent solution (7.5% [w/v] *n*-octyl glucopyranoside, 5 mM cardiolipin in 1 mM diethylenetriamine-pentaacetic acid [DTPA]), and micelles were obtained by bath sonication for 10 min. The kinase reaction was initiated by the addition of 70 μL of a reaction mixture containing 10 μL diacylglycerol (DAG) kinase (GE Healthcare Europe, Munich, Germany), 0.1 M imidazole/HCl (pH 6.6), 0.2 mM DTPA, 70 mM NaCl, 17 mM MgCl_2_, 1.4 mM ethylene glycol tetraacetic acid, 2 mM dithiothreitol, 1 µM adenosine triphosphate (ATP), and 5 μCi [^32^P]γATP. The kinase reaction was performed for 60 min at room temperature with shaking at 300 rpm. The assay was terminated by the addition of 1 mL CHCl_3_:CH_3_OH:1 N HCl (100:100:1, v/v/v), 170 μL buffered saline solution (135 mM NaCl, 1.5 mM CaCl_2_, 0.5 mM MgCl_2_, 5.6 mM glucose, 10 mM HEPES, pH 7.2), and 30 μL of a 100-mM ethylenediaminetetraacetic acid (EDTA) solution. The samples were vortexed, phases were separated, and the lower phase was collected. The samples were then dried, separated on Silica G60 thin-layer chromatography (TLC) plates with chloroform/acetone/methanol/acetic acid/H_2_O (50:20:15:10:5, v/v/v/v/v), and developed with a Fuji phosphoimager. Ceramide amounts were determined by comparison with a standard curve; C_16_ to C_24_ ceramides were used as substrates.

### Transmission electron microscopy

Freshly isolated mouse hippocampus samples were fixed overnight in a solution of 2.5% (v/v) glutaraldehyde plus 2% (v/v) paraformaldehyde in 100 mM sodium cacodylate (pH 7.2) at 4 °C. After the samples had been washed, postfixation was performed in a 1% OsO_4_ solution in 100 mM sodium cacodylate (pH 7.2) at 4 °C. Sections were contrasted with a saturated uranyl acetate solution in 50% ethanol for 15 min, followed by incubation in a 0.5% (w/v) lead citrate solution in distilled water for 7 min. Finally, the samples were analyzed with a Tecnai G2 transmission electron microscope (FEI Company, Hillsboro, OR, USA) operating at 100 kV. Images were acquired with a Veleta camera (Olympus Soft Imaging Solutions, Italy).

### PP2A activity measurements

PP2A activity was measured with a kit from Millipore (Darmstadt, Germany). Isolated hippocampi were homogenized in a tight Dounce glass homogenizer with 20 mM imidazole-HCl (pH 7.0), 2 mM EDTA, 2 mM ethylene glycol tetraacetic acid (EGTA), and 1 mM benzamidine, each with 10 μM aprotinin and leupeptin. Samples were centrifuged at 2000 × *g* for 5 min at 4 ^o^C, the supernatants were collected and incubated with 4 μg anti-PP2A (C subunit, clone 1D6, Millipore) for 30 min with constant rocking, 30 μL Protein A agarose slurry was added, and the samples were incubated for a further 30 min. The immunoprecipitates were washed five times with Tris-HCl buffered saline and once with 50 mM Tris-HCl (pH 7.0) and 100 μM CaCl_2_. The samples were resuspended in 20 μL of the same buffer, and 60 μL of the threonine phosphopeptide K-R-pT-I-R-R (final concentration, 750 μM) dissolved in distilled H_2_O was added. Samples were incubated for 10 min at 30 ^o^C, centrifuged and 60 μL of the supernatant was added to 200 μL malachite green phosphate detection solution. The color was allowed to develop for 15 min and was read at 650 nm. Phosphate concentrations were determined with a standard curve, and enzyme activities were calculated.

### Purification of lysosomes, Golgi bodies, and the endoplasmatic reticulum

The hippocampus was isolated and homogenized with a tight Dounce homogenizer with 40 strokes in extraction buffer (100 mM HEPES, pH 7.8; 10 mM EGTA; and 250 mM KCl; each with 10 μg/mL aprotinin and leupeptin). The samples were centrifuged at 1000 × *g* at 4 ^o^C for 10 min, and the supernatant was collected and centrifuged at 12,000 × *g* at 4 ^o^C for 15 min. The supernatant was collected, and rough ER-enriched microsomes were prepared by precipitation with CaCl_2_. The samples were brought to 7 mM CaCl_2_ under constant stirring; the samples were then stirred at 4 ^o^C for an additional 15 min and centrifuged at 8000 × *g* for 10 min at 4 ^o^C. The pellet was completely homogenized in extraction buffer and used for lipid and protein analysis. Lysosomes were prepared by centrifugation of the brain homogenates at 1000 × *g* at 4 ^o^C for 10 min. The supernatants were collected and centrifuged at 20,000 × *g* for 20 min at 4 ^o^C. The pellets were resuspended in extraction buffer and diluted into a 19% Optiprep density gradient medium (Sigma) with a protein concentration of 7.5 mg/ml. ER and mitochondria were precipitated with 8 mM (final concentration) CaCl_2_. The samples were centrifuged at 5000 × *g* for 15 min at 4 ^o^C, and the supernatants containing lysosomes were used for lipid and protein analysis.

To prepare Golgi bodies, the brain was homogenized in the extraction buffer with a Dounce homogenizer as above, the homogenate was centrifuged for 15 min at 3000 × *g* at 4 ^o^C, the supernatant was collected, sucrose was added to 1.25 M sucrose, and the samples were mixed and purified over a discontinuous gradient of 1.84 M sucrose/sample/1.1 M sucrose/0.25 M sucrose. The samples were ultracentrifuged at 120,000 × *g* for 3 h at 4 ^o^C. The Golgi-enriched phase was removed from the 1.1 M/0.25 M sucrose interphase and used for lipid and protein analysis.

Purity of the samples was tested by blotting aliquots with anti-LAMP1 antibodies (Abcam, Cambridge, UK; #ab24170) for lysosomes, anti-S6K1 antibodies (Santa Cruz Biotechnology Inc., Santa Cruz, CA, USA; #sc8418) for cytoplasm, anti-calreticulin-antibodies (Abcam; #ab2907) for ER, anti-VDAC1 antibodies (Santa Cruz Biotechnology Inc.; sc390996) for mitochondria, and anti-Golga1 antibodies (Sigma; SAB 1410558) for Golgi bodies. All antibodies were diluted 1:1000 for western blotting, which was performed as described below.

### Western blot studies in the hippocampus or PC-12 cells

Mice were sacrificed, the hippocampal area was removed, immediately shock frozen, and homogenized with a tip sonicator in 300 μL 25 mM HEPES, 3% NP40, 0.1% Triton X-100, 10 mM EDTA, 10 mM sodium pyrophosphate, 10 mM sodium fluoride, 125 mM NaCl, and 10 μg/mL aprotinin/leupeptin. PC-12 cells were incubated with the drugs as described below, washed in HEPES/Saline (H/S; 132 mM NaCl, 20 mM HEPES [pH 7.4], 5 mM KCl, 1 mM CaCl_2_, 0.7 mM MgCl_2_, 0.8 mM MgSO_4_), pelleted, and lysed in the same buffer as the hippocampus. Samples were lysed for 5 min at 4 °C and centrifuged at 14,000 rpm for 5 min at 4 °C. The supernatants were added to 5× SDS-Laemmli buffer and boiled, and proteins were separated by 7.5% or 10% sodium dodecyl sulfate polyacrylamide gel electrophoresis (SDS-PAGE). The gels were blotted onto nitrocellulose membranes overnight, blocked in Starting Block Tris-buffered saline (TBS) blocking buffer (ThermoFisherScientific, Darmstadt, Germany; #37542) for 60 min, incubated with antibodies specific for mTOR (Cell Signalling, Frankfurt am Main, Germany; #2983), phospho-mTOR (Cell Signalling Technology; #5536), Ulk (Abcam; #ab128859), phospho-Ulk serine 555 (Cell Signaling Technology; #5869), phospho-Ulk serine 757 (Cell Signaling Technology; #6888), Beclin (Santa Cruz Biotechnology Inc.; #sc11427), phospho-Beclin (Cell Signaling Technology; #84966), PI3-K/Vps34 (Upstate Biotechnology Inc., Darmstadt, Germany; #06–195), phospho-PI3-K/Vps34 (Cell Signaling Technology; #13857), or p62 (Sigma; #P0067). All antibodies were diluted 1:1000-fold in Starting Block (TBS) blocking buffer and were incubated for 60 min at room temperature with the blots. Blots were washed five times in TBS/0.05% Tween 20, incubated for 60 min with alkaline phosphatase (AP)-coupled antibodies directed against the primary antibodies, washed again five times in TBS/0.05% Tween 20, washed twice in alkaline wash buffer, and developed with the CDP-STAR with NitroBlockII Enhancer system (Perkin Elmer, Rodgau, Germany).

### Immunohistochemical analyses

Mice were treated as described above, euthanized, and perfused via the left heart for 2 min with 0.9% NaCl followed by perfusion with 4% paraformaldehyde (PFA) buffered in phosphate-buffered saline (PBS; pH 7.3) for 15 min. Brains were removed, fixed for an additional 36 h in 4% buffered PFA in PBS, embedded in paraffin, serially sectioned, dewaxed, incubated for 30 min with pepsin (Digest All, Invitrogen, Darmstadt, Germany) at 37 °C, washed, and blocked for 10 min with PBS, 0.05% Tween 20, and 5% fetal calf serum (FCS). The samples were washed again in H/S and were then immunostained for 45 min with antibodies specific for Ulk, phospho-Ulk serine 555, phospho-Ulk serine 757, Beclin, phospho-Beclin, PI3-K/Vps34, phospho-PI3-K/Vps34, or p62. All antibodies were diluted 1:100 in H/S + 1% FCS. The samples were washed 3 times in PBS + 0.05% Tween 20, washed once in PBS, and incubated for 45 min with Cy3-coupled donkey anti-rabbit immunoglobulin G (IgG) F(ab)_2_ fragments (Jackson ImmunoResearch, Newmarket, UK). The specimens were then washed again three times in PBS plus 0.05% Tween 20 and once in PBS. Finally, the sections were embedded in Mowiol and analyzed with a LEICA TCS SL confocal microscope (Leica, Mannheim, Germany). Control stainings with isotype-matched control antibodies showed very weak or no signals and served as specificity controls.

To ensure equal thickness of the slides, we also stained sections with anti-actin and antibodies. To this end, five slides from each genotype and treatment were dewaxed, incubated for 30 min with pepsin, washed, and blocked as above. Samples were stained for 45 min with antibodies specific for actin (Santa Cruz Inc.; #47778) and then washed and incubated for 45 min with Cy3-coupled donkey anti-rabbit IgG F(ab)_2_ fragments (Jackson ImmunoResearch). The specimens were then processed as above and analyzed for fluorescence using conventional fluorescence microscopy (Supplementary Fig. 9).

All confocal microscopy studies were quantified by measuring the fluorescence in 20 cells per sample at the neurogenetic zone of the dentate gyrus (a total of 80 or 120 cells per group).

### Immunohistochemical bromodeoxyuridine and doublecortin stainings

For BrdU staining, mice were injected with BrdU four times every 2 h. Mice were sacrificed 24 h after the first injection, and brains were prepared as above. Paraffin-embedded sections were dewaxed, treated for 20 min with pepsin at 37 °C, washed, incubated for 2 h with 50% formamide in 300 mM NaCl and 30 mM sodium citrate (pH 7.0) at 65 °C, and washed twice in sodium citrate buffer. The DNA was denatured for 30 min at 37 °C with 2 M HCl, washed, neutralized for 10 min with 0.1 M borate buffer (pH 8.5), washed, and blocked with 0.05% Tween 20 and 5% FCS in PBS (pH 7.4). The samples were then stained with 5 μg/mL BrdU-specific antibodies (Roche, Mannheim, Germany, #111703760001) for 45 min at 22 °C, washed, and stained with Cy3-coupled F(ab)_2_ anti-mouse IgG (Jackson ImmunoResearch).

Neuronal maturation was analyzed by doublecortin staining. To this end, the specimens were dewaxed and rehydrated. The samples were then demasked by microwave treatment for 15 min at 650 W in citrate buffer, cooled, washed in PBS, blocked in blocking solution (CANDOR Bioscience, Wangen, Germany; #110125) and stained with antibodies against doublecortin (1:1000; Abcam, Cambridge, UK) for 45 min at 22 °C. The specimens were washed three times in PBS/0.05% Tween 20 and once in PBS, stained with Cy3-anti-rabbit F(ab)_2_ fragments for 45 min at room temperature, washed again three times in PBS/0.05% Tween 20 and once in PBS, and finally embedded in Mowiol.

All sections were analyzed with a LEICA TCS SL fluorescence microscope. Every tenth section of serial sections of the hippocampus was counted by an investigator blinded to the nature of the samples.

### PC-12 cells

PC-12 cells were cultured in minimum essential medium (MEM) supplemented with 10% horse serum, 10 mM HEPES (pH 7.3), 10 mM penicillin/streptomycin, 1 mM sodium pyruvate, 2 mM l-glutamine, and 0.05 mM β-mercaptoethanol. The cells were incubated for 14 days with 0.25 μM amitriptyline, 0.5 μg/mL corticosterone, a combination of amitriptyline and corticosterone, or left untreated. D609 (25 μg/mL) was added to the cells for 3 days as single treatment or in combination with corticosterone (0.5 μg/mL). Medium was changed every 6 h for D609 or every 12 h for all other treatments. Controls were left untreated. Cells were finally washed twice in H/S; lysed in 25 mM HEPES, 3% NP40, 0.1% Triton X-100, 10 mM EDTA, 10 mM sodium pyrophosphate, 10 mM sodium fluoride, 125 mM NaCl, and 10 µg/mL aprotinin/leupeptin; and further processed for Western blotting as described above. For immunostaining of PC-12 cells, the cells were treated as above, immobilized on poly-l-lysine (Sigma) coated glass coverslips for 60 min, washed in H/S, and fixed for 15 min in 1% PFA buffered with PBS (pH7.4). The samples were washed three times in H/S, permeabilized for 5 min in 0.1% Triton X-100 in PBS, washed in H/S, and stained as described above for the tissue sections.

### Transfection of RFP-62, RFP–Lc3B, and RFP–GFP–Lc3B

Transfection was performed according to the instructions of the vendor (Invitrogen, Darmstadt, Germany) using a Baculovirus system with a mammalian promoter. PC-12 cells were grown on glass coverslips in 24-well plates for microscopy studies or in 12-well plates in suspension for flow cytometry. The cells were treated with corticosterone (0.5 μg/mL) for 14 days, with amitriptyline (0.25 μM) for 12 days, with the combination of corticosterone (14 days) and amitriptyline (12 days), with D609 (25 μg/mL) for 3 days, or with the combination of corticosterone (14 days) and D609 (3 days); control cells were left untreated. The gene constructs were added to the cells at a multiplicity of infection (MOI) of 30 viral particles per cell, and infected cells were analyzed after 24 h. We determined the number of red dots per cell in 20 cells per sample from six independent transfections (a total of 120 cells) to measure p62 and Lc3B concentrations in vesicular structures. To determine the fusion of autophagosomes with lysosomes, i.e., the formation of autophagolysosomes, we transfected PC-12 cells with a baculovirus tandem sensor RFP–GFP–Lc3B (Invitrogen) at an MOI of 30 viral particles per cell. The fluorescence of GFP is pH sensitive and is quenched upon acidification, whereas the fluorescence of RFP is pH insensitive. The samples were analyzed by flow cytometry for determining the percentage of phagolysosomes (high RFP-fluorescence, low GFP-fluorescence) in the total number of phagosomes (high RFP- and GFP-fluorescence).

### Behavioral studies

Behavioral testing was performed between 3:00 p.m. and 6:00 p.m. under diffuse indirect room light. All tests were performed on separate days. If appropriate, animals were tracked by a video camera (Noldus Systems, Worpswede, Germany). For the novelty-suppressed feeding test (latency to feed test), mice were fasted for 24 h. We then recorded the time during which the mice explored a new environment before they began eating. For the light/dark box test, mice were placed in a dark and safe compartment that was connected via a 5- × 5-cm rounded-corner aperture to an illuminated, open, and thus aversive area. The time that the mouse spent in each of the separate compartments was recorded. In the open-field arena test, the mice were released near the wall of a 50- × 50-cm white plastic cage with sidewalls 30 cm high. Animals were observed for 30 min, and the time during which the animal was more than 10 cm away from the wall was recorded. In the coat state test, the appearance of the coat (groomed vs unkempt coat) was scored on the head, neck, back, and ventrum with either a zero for a normal status or 1 for an unkempt status. In the splash test, 200 µL of a 10% sucrose solution was spotted onto the mouse’s snout, and the latency to begin grooming and the grooming frequency over 5 min were measured. Control experiments showed that the various genotypes did not differ in their locomotion.

### Quantification and statistical analysis

Data are expressed as arithmetic means ± SD. For the comparison of continuous variables from independent groups with one variable (treatment), we used one-way ANOVA followed by post hoc Student’s *t*-tests for all pairwise comparisons, applying the Bonferroni correction for multiple testing. The *P* values for the pairwise comparisons were calculated after Bonferroni correction. All values were normally distributed and the variances were similar. For the analysis of groups with two variables (treatment and genotype) we used two-way ANOVA. A *P* value of 0.05 or less (two-tailed) was considered statistically significant. The sample size planning was based on the results of two-sided Wilcoxon–Mann–Whitney tests (free software: G*Power, Version 3.1.7, University of Duesseldorf, Germany). Investigators were blinded for histology experiments and animal identity. Prior to the experiments animals were randomly assigned to cages by a technician, who was not involved in the experiments. Cages were randomly assigned to the different experimental groups. Western blots and fluorescence or confocal microscopy stainings were quantified with Image J software (National Institutes of Health, Bethesda, MD, USA), and results are expressed as arbitrary units (a.u.) or as relative arbitrary units. To determine relative units, a randomly chosen control was set at 100%, and all other values were calculated in relation to this sample. In fluorescence and confocal microscopy studies, 20 or 25 randomly chosen cells per sample were quantified, resulting in the analysis of a total of 100–150 cells. These values were averaged and are given as means ± SD in arbitrary units.

## Electronic supplementary material

Abbreviations

Supplementary Legends

Supplementary Figures
