# Supplementary material for: Antidepressants act by inducing autophagy controlled by sphingomyelin–ceramide
Source: Mol Psychiatry. 2018 Jul 23;23(12):2324–46. doi: 10.1038/s41380-018-0090-9 (PMC6294742; doi:10.1038/s41380-018-0090-9)
Supplement: Supplementary file 1 — Abbreviations [file 41380_2018_90_MOESM1_ESM.pdf]

Abbreviation list

ANOVA: Analysis of variance
Asm: Acid sphingomyelinase
BrdU: Bromodeoxyuridine
CUS: Chronic unpredictable environmental stress
D609: Tricyclodecan-9-yl-xanthogenate
ER: Endoplasmic reticulum
GFP: Green fluorescent protein
HDL: High-density lipoprotein
H/S: HEPES/Saline
IgG: Immunoglobulin
Lc3B: Microtubule-associated protein light-chain3
mTOR: Mammalian target of rapamycin
MDD: Major depressive disorder
P62: SQSTM1 (sequestosome 1
PBS: Phosphate buffered saline
PI3-K: Phosphatidylinositol 3-kinase
PFA: Paraformaldehyde
PP2A: Phosphatase 2A
RFP: Red fluorescent protein
Smpd1: Sphingomyelin phosphodiesterase 1
TEM: Transmission electron microscopy
Ulk: Unc-51 like autophagy activating kinase
Vps34: Phosphatidyl 3-kinase VPS34 (EC:2.7.1.137), Vacuolar protein sorting-associated protein 34
wt: Wildtype

Legends:
Ami: Amitriptyline
Ap:Autophagosome
Aly: autophagolysosome
Ax: Myelin-surrounded axon
Cort: Corticosterone

Fluo: Fluoxetine

M: Mitochondria

N: Nucleus

OA: Okadaic acid

Sp: Spautin-1
