# Supplementary material for: Antidepressants act by inducing autophagy controlled by sphingomyelin–ceramide
Source: Mol Psychiatry. 2018 Jul 23;23(12):2324–46. doi: 10.1038/s41380-018-0090-9 (PMC6294742; doi:10.1038/s41380-018-0090-9)
Supplement: Supplementary file 2 — Supplementary Legends [file 41380_2018_90_MOESM2_ESM.docx]

**Supplementary Figures**

Anne Gulbins1*, Fabian Schumacher1,2*, Katrin Anne Becker1*, Barbara Wilker1, Matthias Soddemann1, Francesco Boldrin3, Christian P. Müller4, Michael J. Edwards5, Michael Goodman5, Charles C. Caldwell5, Burkhard Kleuser2, Johannes Kornhuber4, Ildiko Szabo3, Erich Gulbins1,5

# Supplementary Figure 1:

The schematic drawing visualizes the time course of the treatments.

# Supplementary Figure 2: Tricyclodecan-9-yl-xanthogenate does not inhibit acid sphingomyelinase activity and does not change lysosomal sphingomyelin in the hippocampus *in vivo*

It might be possible that D609 acts by inhibition of the phosphatidylcholine-specific phospholipase (PC-PLC). However, PC-PLC has not been cloned and studies by Luberto and Hannun suggest that sphingomyelin synthase and PC-PLC are identical (Luberto, C. & Hannun Y.A. Sphingomyelin synthase, a potential regulator of intracellular levels of ceramide and diacylglycerol during SV40 transformation. J. Biol. Chem. **273**, 14550-14559, 1998). However, D609 mediated inhibition of PC-PLC might reduce the release of diacylglycerol (DAG) and phosphocholine. DAG might activate the Asm and a reduction of DAG might finally result in reduced lysosomal Asm activity and sphingomyelin accumulation. To exclude this possibility, we determined Asm activity (**a**) in hippocampus extracts from mice treated with D609 and (**b**) sphingomyelin in isolated lysosomes from hippocampus extracts.

(**a**) Treatment with tricyclodecan-9-yl-xanthogenate (D609) does not inhibit Asm activity in the hippocampus. Mice were treated with D609 or left untreated for 5 days. The hippocampus was removed, homogenized with a tip sonicator in 250 mM sodium acetate (pH 5.0) and 0.1% NP-40, after which 50 nCi [14C]-sphingomyelin per sample (Perkin Elmer, Waltham, MA, USA; 52 mCi/mmol) was added. The substrate was

dried, resuspended in 250 mM sodium acetate (pH 5.0) and 0.1% NP-40, and bath

sonicated for 10 min before addition to the samples. Samples were then incubated for 60 min at 37°C, the reaction was stopped by the addition of 800 μL chloroform/methanol (2:1, v/v), phases were separated by centrifugation, and aliquots of the upper phase containing the released [14C]-phosphorylcholine were removed and quantified by liquid scintillation. Treatment with amitriptyline served as a positive control. Shown are the means ± SD; n = 5 each; ***p < 0.001, Anova.

(**b**) D609 does not chnage lysosomal sphingomyelin concentrations measured by mass spectrometry of purified lysosomes from the hippocampus indicating that D609 does not act via PC-PLC in the present studies. Shown are the means ± SD; n = 5 each; *p < 0.05, Anova.

# Supplementary Figure 3: Purification of organelles

Western blots from purified lysosomes (left), Golgi bodies (middle), or endoplasmic reticulum (ER; right) show a high degree of purification of these organelles. Aliquots of purified organelles were separated by sodium dodecyl sulfate polyacrylamide gel electrophoresis (SDS-PAGE), and the lanes were blotted as indicated with anti- Lamp1- to detect lysosomes, anti-Golga1- to detect Golgi bodies, anti-calreticulin- to detect ER, anti-S6K- to detect cytoplasm, and anti-VDAC1-antibodies to detect mitochondria. Shown is a representative blot from the 5 independent experiments.

**Supplementary Figure 4: Fluoxetine, amitriptyline and D609 increase ceramide concentrations in the endoplasmic reticulum (ceramide kinase assays)** Fluoxetine, amitriptyline or D609 treatment of wild-type (wt) mice for 12 days (fluoxetine and amitriptyline) or 3 days (D609) increases ceramide concentrations in the endoplasmic reticulum (ER), whereas fluoxetine and amitriptyline have no effect in Asm-deficient mice. Mice were sacrificed after the indicated treatment, the hippocampus was removed, the ER was isolated, and ceramide concentrations were measured with the ceramide kinase method. Shown are the means ± SD; n = 5 each;

*p<0.05, **p < 0.01, ***p < 0.001, ANOVA.

**Supplementary Figure 5: Expression levels of protein phosphatase 2A, Ulk, Beclin, and PI3-K/Vps34 do not change after treatment with antidepressants** Expression of protein phosphatase 2A (PP2A) (**a**), Ulk (**b**), Beclin (**c**), and PI3- K/Vps34 (**d**) does not change after treatment of wild-type mice with amitriptyline,

fluoxetine, or tricyclodecan-9-yl-xanthogenate (D609) with or without corticosterone or chronic unpredictable environmental stress (CUS), as compared to untreated mice. Mice were treated as indicated; hippocampi were isolated and homogenized; and Western blots of the extracts were performed. Protein concentrations were determined and equal amounts of the samples were loaded onto the gels. Aliquots were blotted for actin (lower blots in Fig. 2). Blots were quantified with Image J software and normalized to actin. Shown are the means ± SD of the quantified bands from 5 independent experiments; *p <0.05, **p < 0.01, ***p < 0.001, ANOVA.

# Supplementary Figure 6: Quantification of Western blots

(**a-f**) The confocal fluorescence microscopy studies displayed in Fig. 3 were analyzed for intensity of the fluorescence signal using Image J. Shown are the means ± SD from 6 independent studies; *p <0.05, **p < 0.01, ***p < 0.001, ANOVA. Cort- Corticosterone; CUS-chronic unpredictable environmental stress.

# Supplementary Figure 7: Amitriptyline, fluoxetine, and tricyclodecan-9-yl- xanthogenate do not affect expression or phosphorylation of mammalian target of rapamycin

(**a**) Phosphorylation of mammalian target of rapamycin (mTOR) is not affected by treatment with amitriptyline, fluoxetine, or tricyclodecan-9-yl-xanthogenate (D609) and does not differ between wt, Asm-deficient, and Asm-transgenic mice.

(**b**) Controls confirmed that the expression levels of mTOR did not differ between treated or untreated mice or between the various mouse strains.

Phosphorylation and expression of mTOR were determined by Western blots of extracts prepared from isolated hippocampi. Protein content and loading of the samples were controlled by Western blotting for actin. Shown are the means ± SD of the results of the quantification of 5 independent Western blots/per group using Image J software; *p <0.05, **p < 0.01, ***p < 0.001, ANOVA.

# Supplementary Figure 8: Quantification of fluorescence stainings

(**a-d**) We quantified the fluorescence in 20 cells per section at the neurogenetic zone of the dentate gyrus (a total of 120 cells per group). The fluorescence of p-Ulk in the 1st randomly chosen section from untreated wildtype mice was set at 100% and all other samples were analyzed based on this value. In addition, we quantified the

fluorescence in 20 cells per section that were incubated with isotype control antibodies (Cy3-F(ab)2-fragments of donkey anti rabbit IgG) to the primary antibodies (**d**). Please note that the unspecific staining is less than 5% of the specific signal. The fluorescence intensity was quantified with Image J software. Shown are the means ± SD; n = 6; *p < 0.05, **p < 0.01, ***p < 0.001, ANOVA.

# Supplementary Figure 9: Control for thickness of sections

To test for equal thickness of the slides, we stained sections with Cy3-coupled anti- actin-antibodies. The specimens were processed as above and analyzed for fluorescence using conventional fluorescence microscopy. We analyzed the fluorescence in equally-sized areas of the cell body from 20 neurons per section in the neurogenetic zone of the dentate gyrus from 5 mice/group (a total of 100 cells per

group). The fluorescence of actin in the 1st randomly chosen section from untreated wildtype mice was set at 100% and the other samples were analyzed based on this value. Shown are the means ± SD of the fluorescence; *p <0.05, **p < 0.01, ***p < 0.001, ANOVA.

# Supplementary Figure 10: Topology of ceramide

(**a**) PC-12 cells were left untreated; were treated with 0.5 μg/mL corticosterone, 0.25

μM amitriptyline, 0.25 μM amitriptyline plus 10 nM okadaic acid (an inhibitor of protein phosphatase 2 [PP2A]), 0.5 μg/mL corticosterone plus 0.25 μM amitriptyline,

0.5 μg/mL corticosterone plus 0.25 μM amitriptyline plus 10 nM okadaic acid for 14 days; or were treated with 25 μg/mL tricyclodecan-9-yl-xanthogenate (D609), 25

μg/mL D609 plus 10 nM okadaic acid, or 25 μg/mL D609 plus 0.5 μg/mL corticosterone plus 10 nM okadaic acid for 3 days. Cells were lysed, and Western blots were performed for phosphorylation of Ulk and Beclin. Western blots for actin served to normalize the samples. Western blots were quantified with Image J software. Controls measured the activity of PP2A and confirmed inhibition of the enzyme by okadaic acid. The results indicate that PP2A activation is required for phosphorylation of Ulk and Beclin. Shown are the means ± SD; n = 6; *p < 0.05, **p < 0.01, ***p < 0.001, ANOVA.

(**b**) Ceramide in the endoplasmic reticulum (ER) is increased after treatment with amitriptyline or D609 plus corticosterone. PC-12 cells were stained with Cy3-coupled anti-ceramide- and fluorescein isothiocyanate (FITC)-labelled anti-Lamp1-, FITC-anti-

calreticulin-, or FITC-anti-β1-integrin-antibodies. The studies showed an intracellular accumulation of ceramide in the ER, a reduction of lysosomal ceramide concentrations after treatment with amitriptyline, and an increase in ceramide concentrations in the ER after D609 treatment, but no change in ceramide concentrations in the plasma membrane after treatment with amitriptyline or D609. In addition, we stained intact PC-12 cells with Cy3-coupled anti-ceramide antibodies and did not detect an increase in extracellularly oriented ceramide concentrations in stressed or unstressed cells treated with amitriptyline (not shown). FITC-Lamp-1- positive, FITC-calreticulin-positive, and FITC-β1-integrin-positive areas were randomly chosen, and Cy3-ceramide fluorescence was quantified in 3 areas of interest per cell in a total of 20 cells per sample and 4 independent experiments (a total of 240 areas of interest in 80 cells). Shown are the means ± SD of the fluorescence; *p <0.05, **p < 0.01, ***p < 0.001, ANOVA.

Methods: PC-12 cells were cultured on coverslips, treated as indicated, or left untreated. The medium was removed, and cells were immediately fixed in 1% paraformaldehyde (Sigma) in phosphate-buffered saline (PBS; pH 7.4) for 10 min. Cells were washed 3 times with PBS, permeabilized with 0.1% Triton X-100 in PBS for 5 min at room temperature or left intact, washed again, and incubated for 15 min with 5% fetal calf serum (FCS) in HEPES/saline (H/S) buffer consisting of 132 mM NaCl, 20 mM HEPES (pH 7.4), 5 mM KCl, 1 mM CaCl2, 0.7 mM MgCl2, and 0.8 mM

MgSO4 to block non-specific binding. Cells were then washed again; consecutively

incubated for 45 min with anti-ceramide- (diluted 1:100, mouse monoclonal IgM; Glycobiotech), anti-Lamp-1- (1:200 dilution; Abcam; #24245), anti-calreticulin- (1:100 dilution; Abcam; #ab2907), or anti-β1-integrin-antibodies (1:100 dilution; Merck Millipore; clone MB1.2, #MAB1997); and diluted in H/S supplemented with 1% FCS. The samples were washed 3 times with 0.05% Tween-20/PBS for 5 min between and after the incubations. Primary antibodies were visualized by a 45-min incubation with FITC- or Cy3-conjugated F(abʹ)2 fragments (Jackson ImmunoResearch). Finally the samples were washed again 3-times with 0.05% Tween 20/PBS and once in PBS, mounted with Mowiol (Kuraray Specialities Europe GmbH), and analyzed with a Leica TCS SP5 confocal microscope and Leica LCS software (Leica Microsystems). In addition, intact cells were stained with anti-ceramide antibodies and secondary Cy3-coupled anti-IgM F(ab)2 fragments and analyzed by confocal microscopy as above.

(**c**) Sphingomyelin did not activate PP2A, whereas C16-ceramide induced a marked activation of the enzyme. PC-12 cells were lysed, and the lysates were incubated with 10 μM C16-ceramide or 10 μM sphingomyelin. The lipids were solubilized in 0.01% octyl glucopyranoside micelles. PP2A was then immunoprecipitated, and the activity was measured as described in the method section.

# Supplementary Figure 11: Tricyclodecan-9-yl-xanthogenate acts against major depressive disorder within 36 to 72 hr

Mice were stressed with corticosterone for 15 days and treated for 36 hr or 72 hr with tricyclodecan-9-yl-xanthogenate (D609) before the behavior of the mice was measured. The results show that D609 improved behavior already after 36 hr of treatment, although a complete reversal of stressed behavior required 3 days of treatment with D609. Shown are the means ± SD of the fluorescence; *p <0.05, **p < 0.01, ***p < 0.001, ANOVA.
