# Supplementary material for: Antidepressants act by inducing autophagy controlled by sphingomyelin–ceramide
Source: Mol Psychiatry. 2018 Jul 23;23(12):2324–46. doi: 10.1038/s41380-018-0090-9 (PMC6294742; doi:10.1038/s41380-018-0090-9)

Supplementary Fig. 1

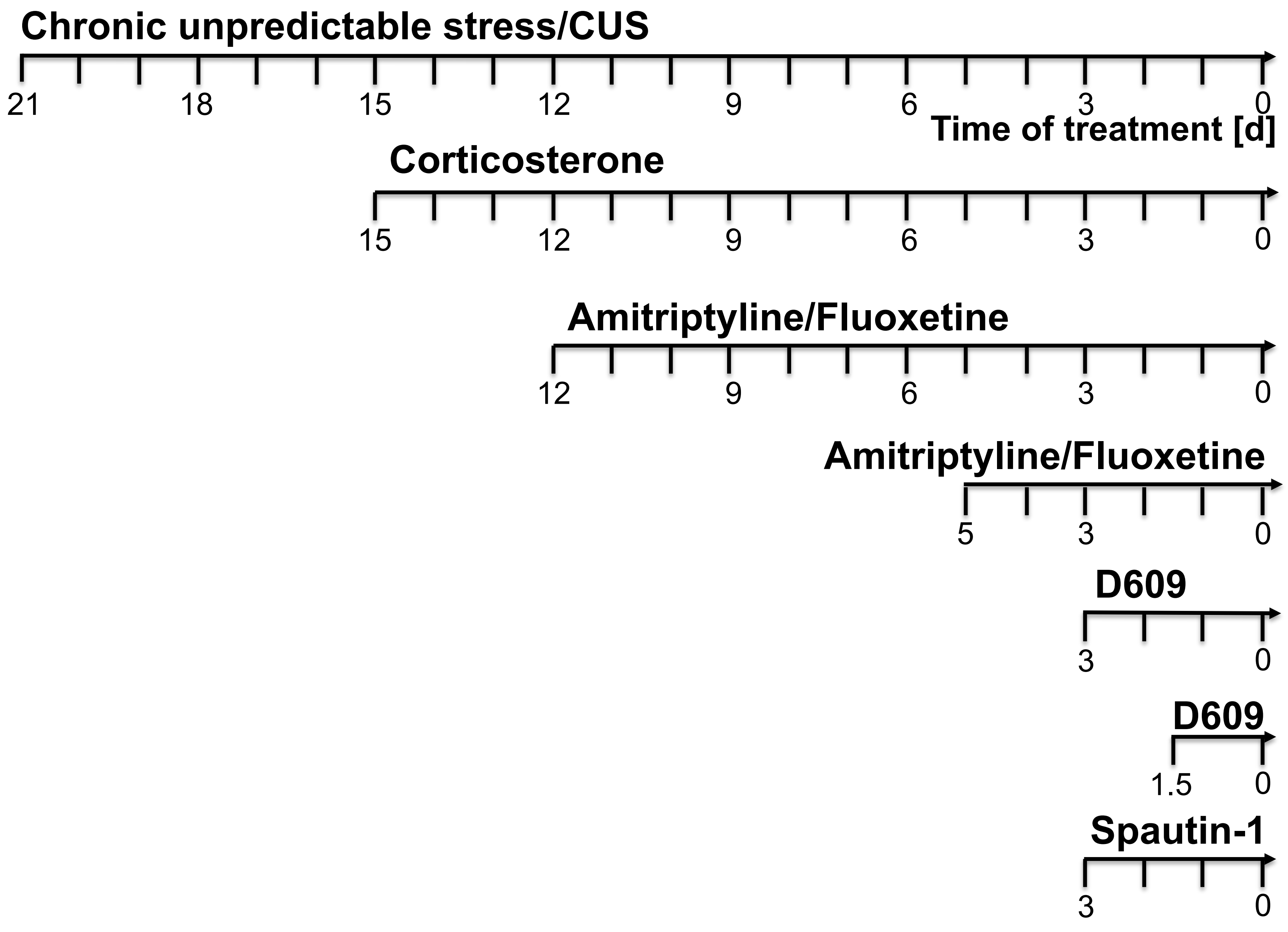

Supplementary Figure 2

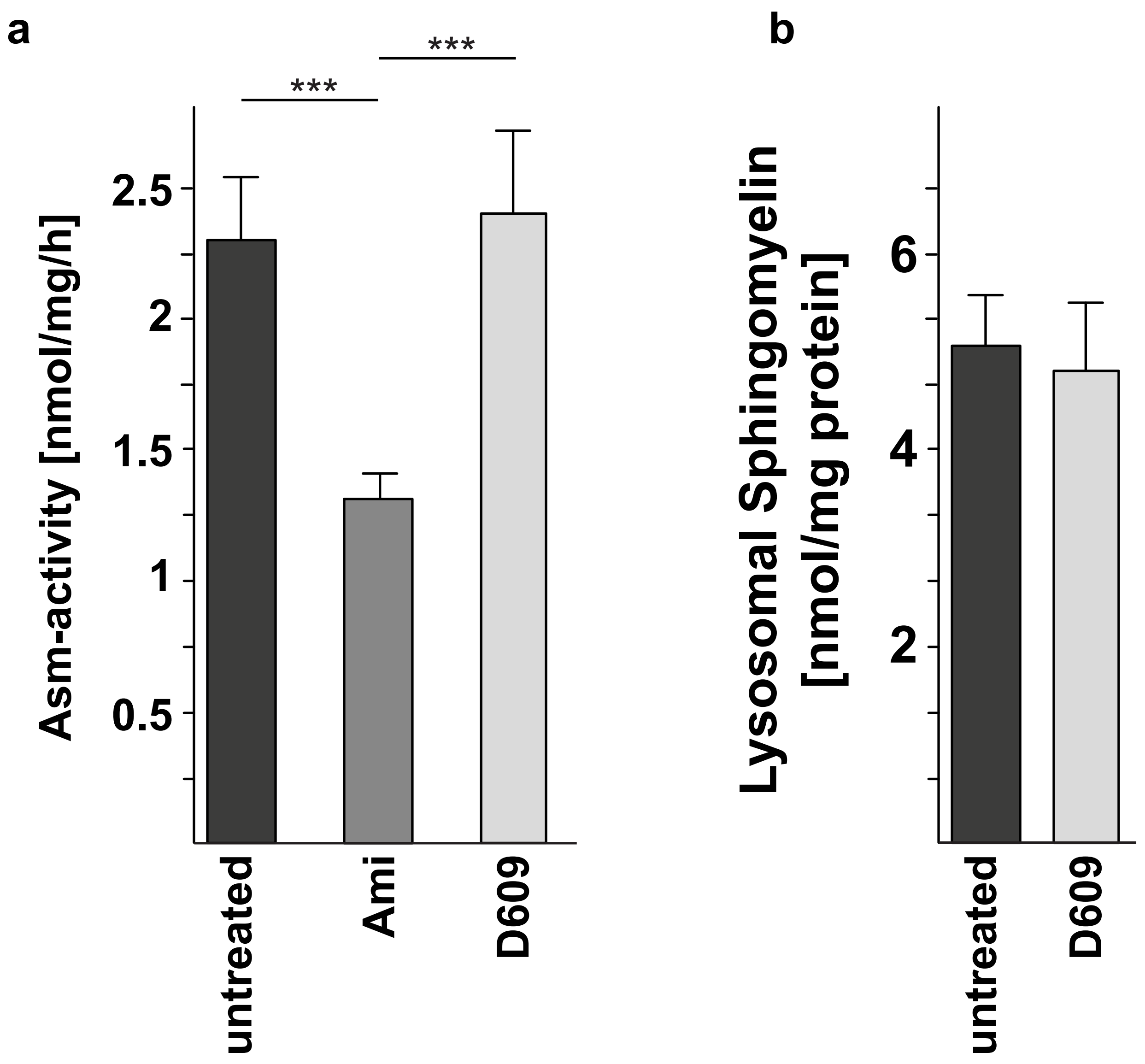

Supplementary Fig. 3

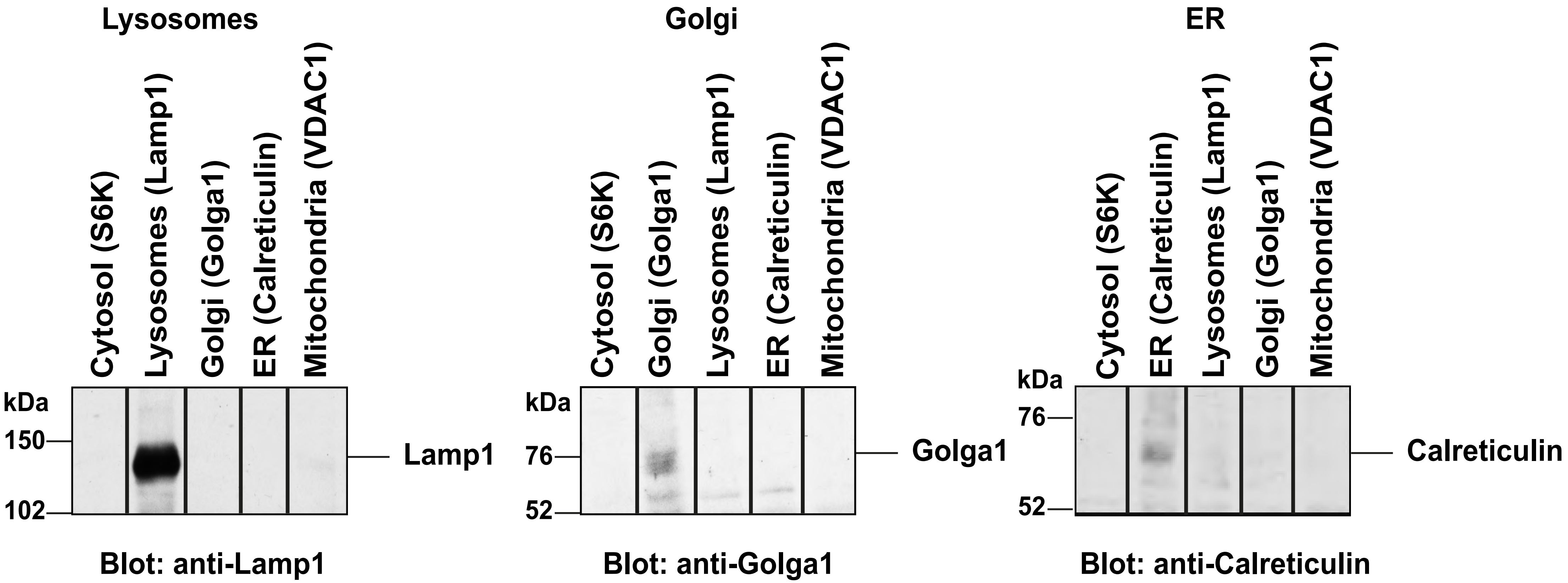

Supplementary Fig. 4

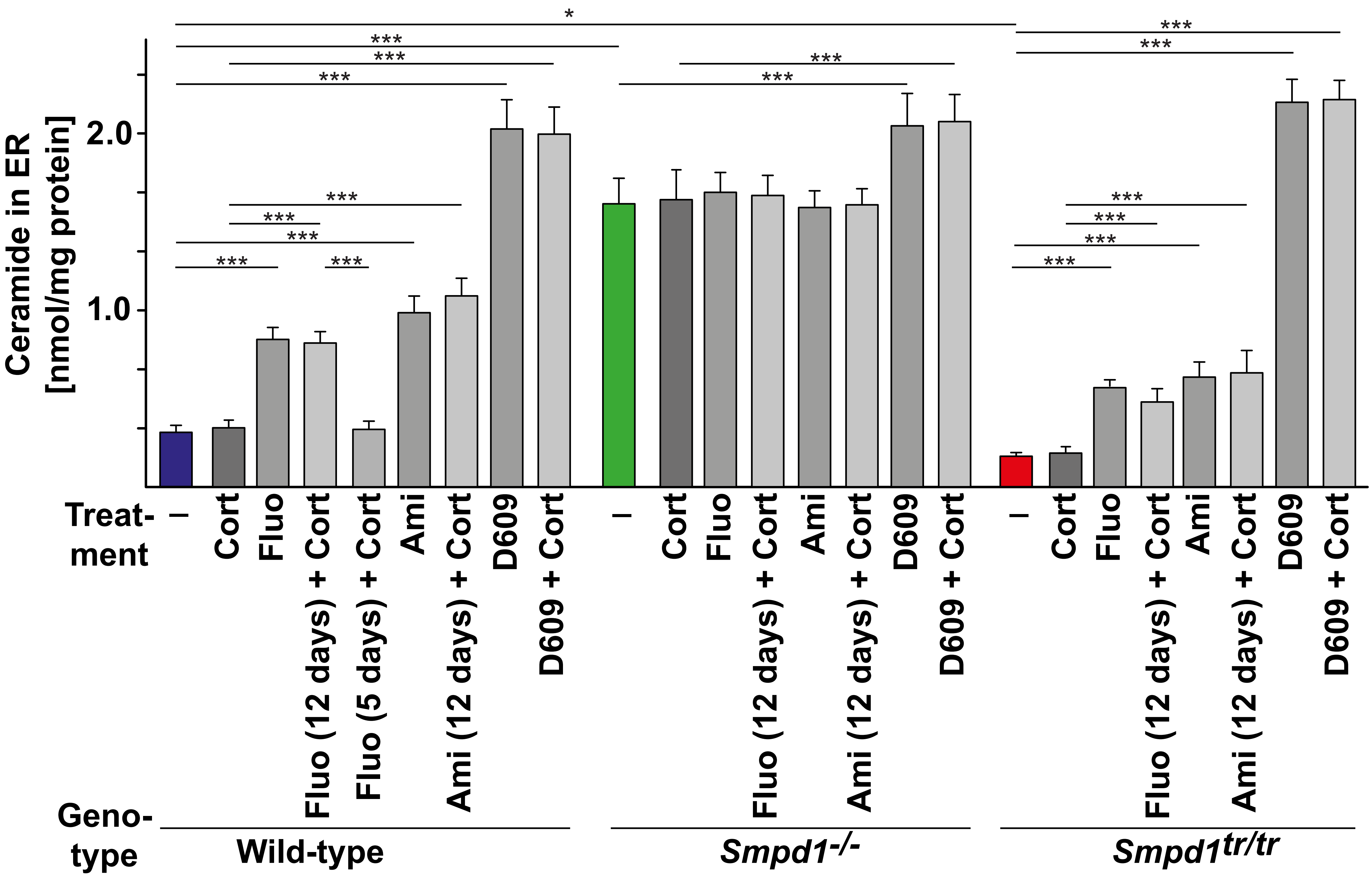

Supplementary Fig. 5a

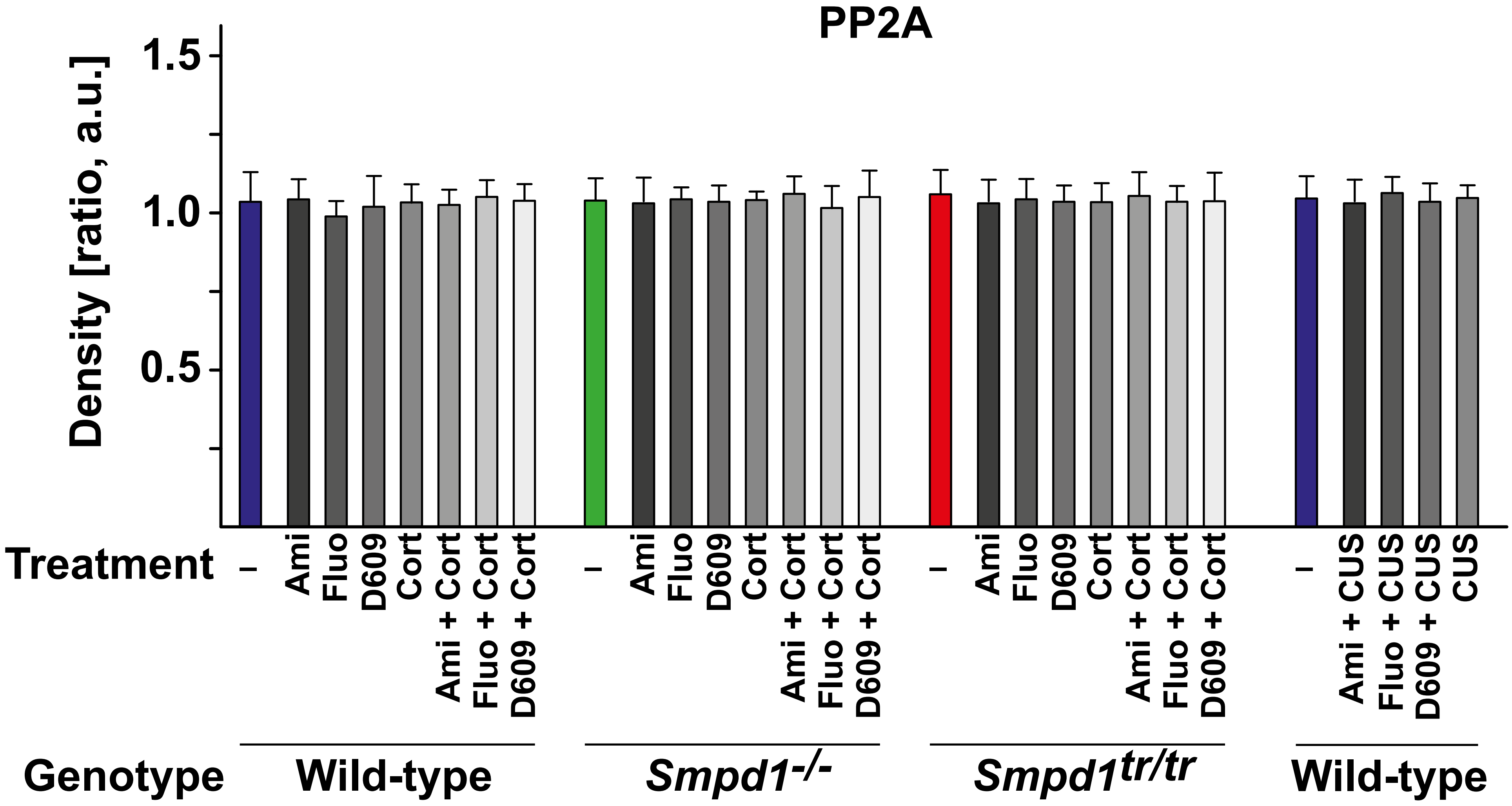

Supplementary Fig. 5b

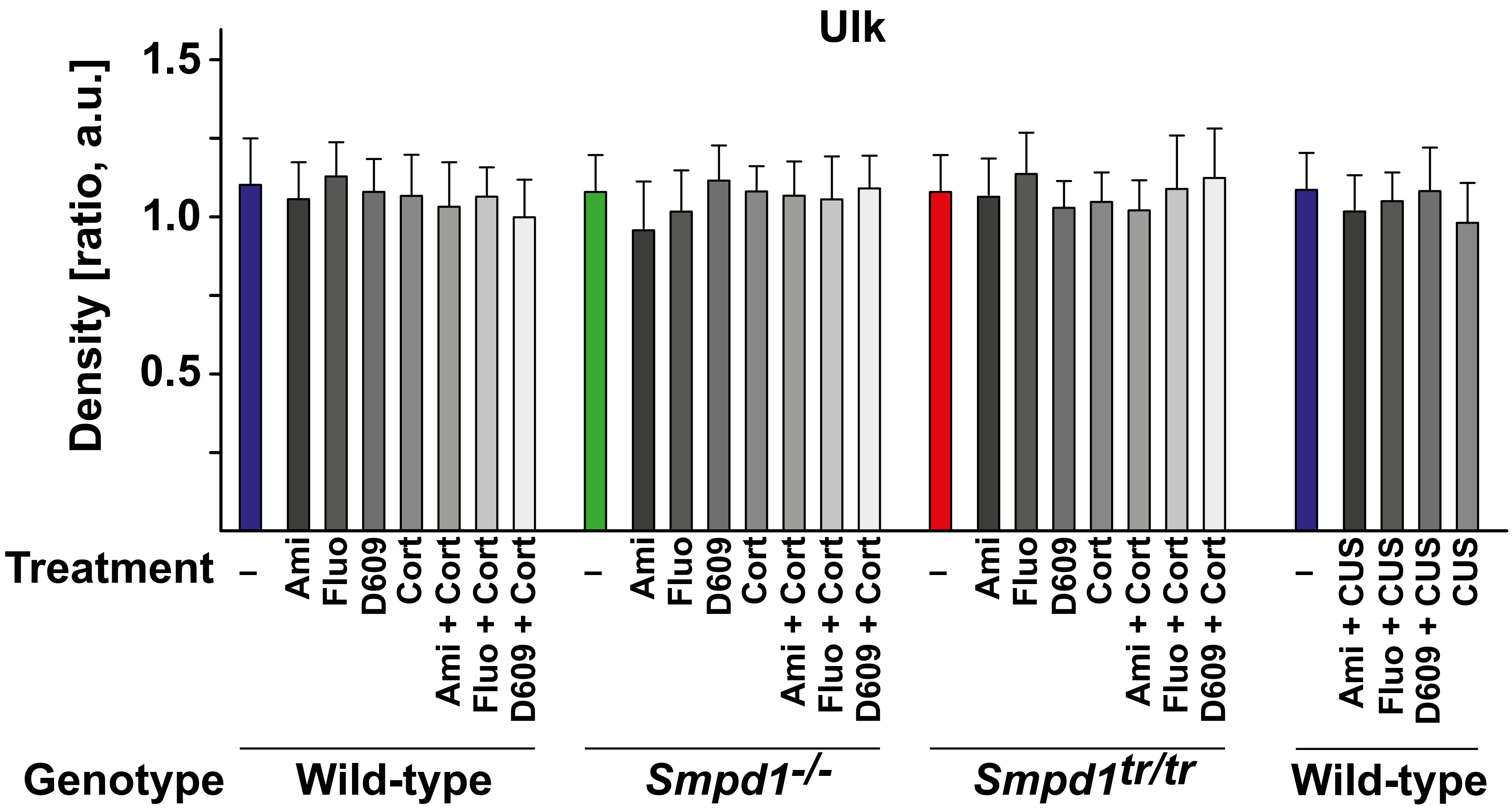

Supplementary Fig. 5c

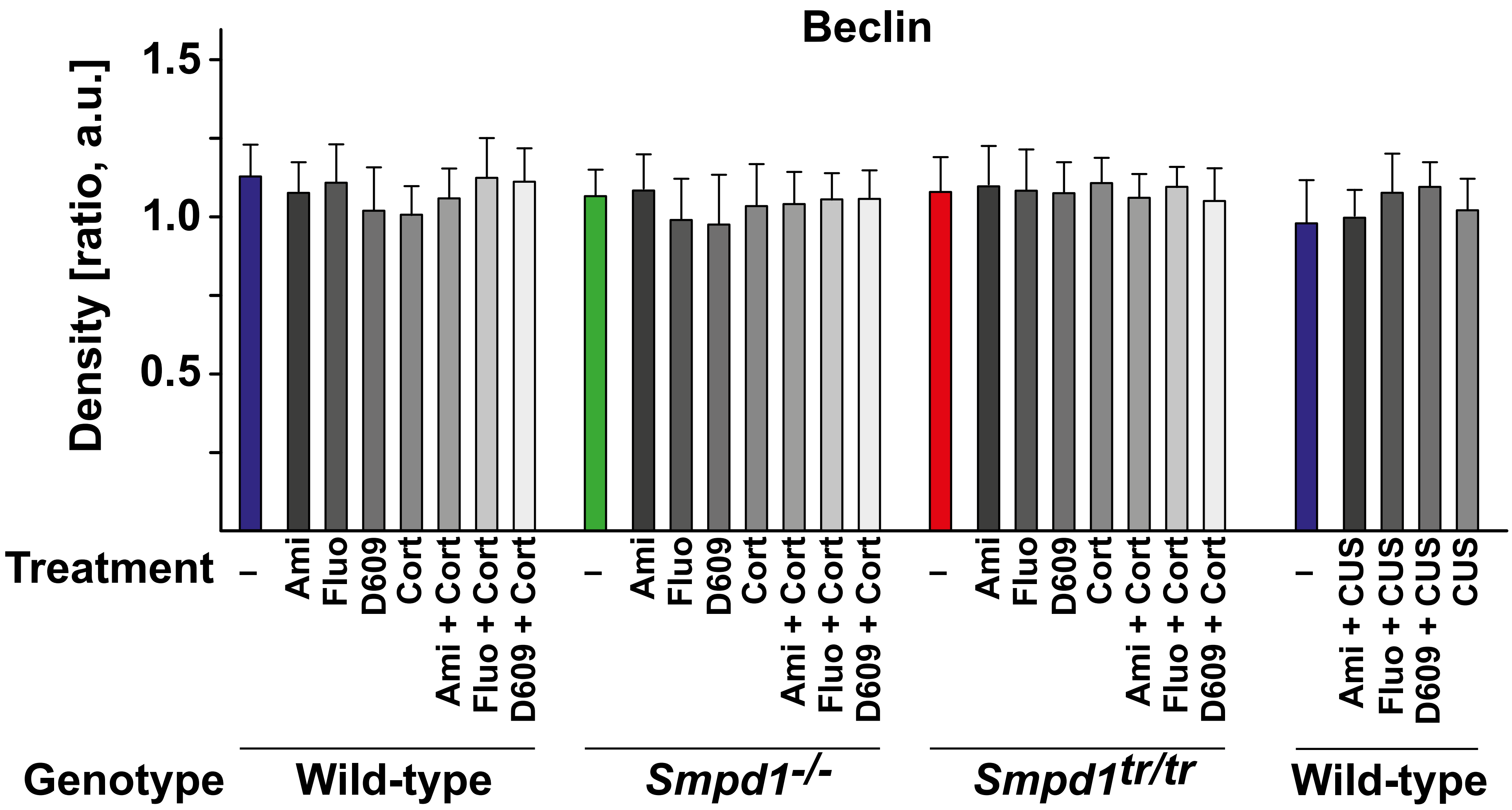

Supplementary Fig. 5d

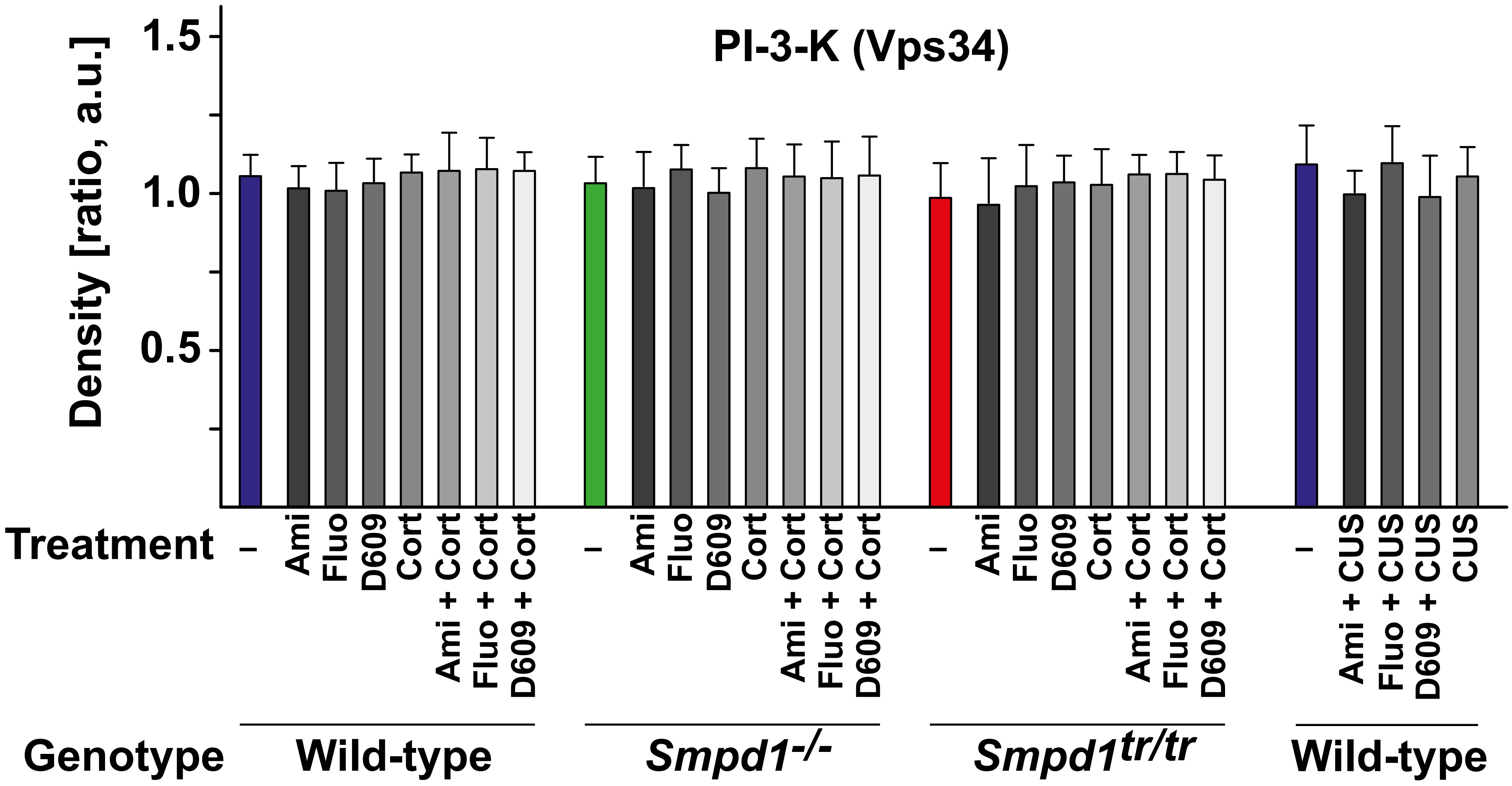

Supplementary Fig. 6a

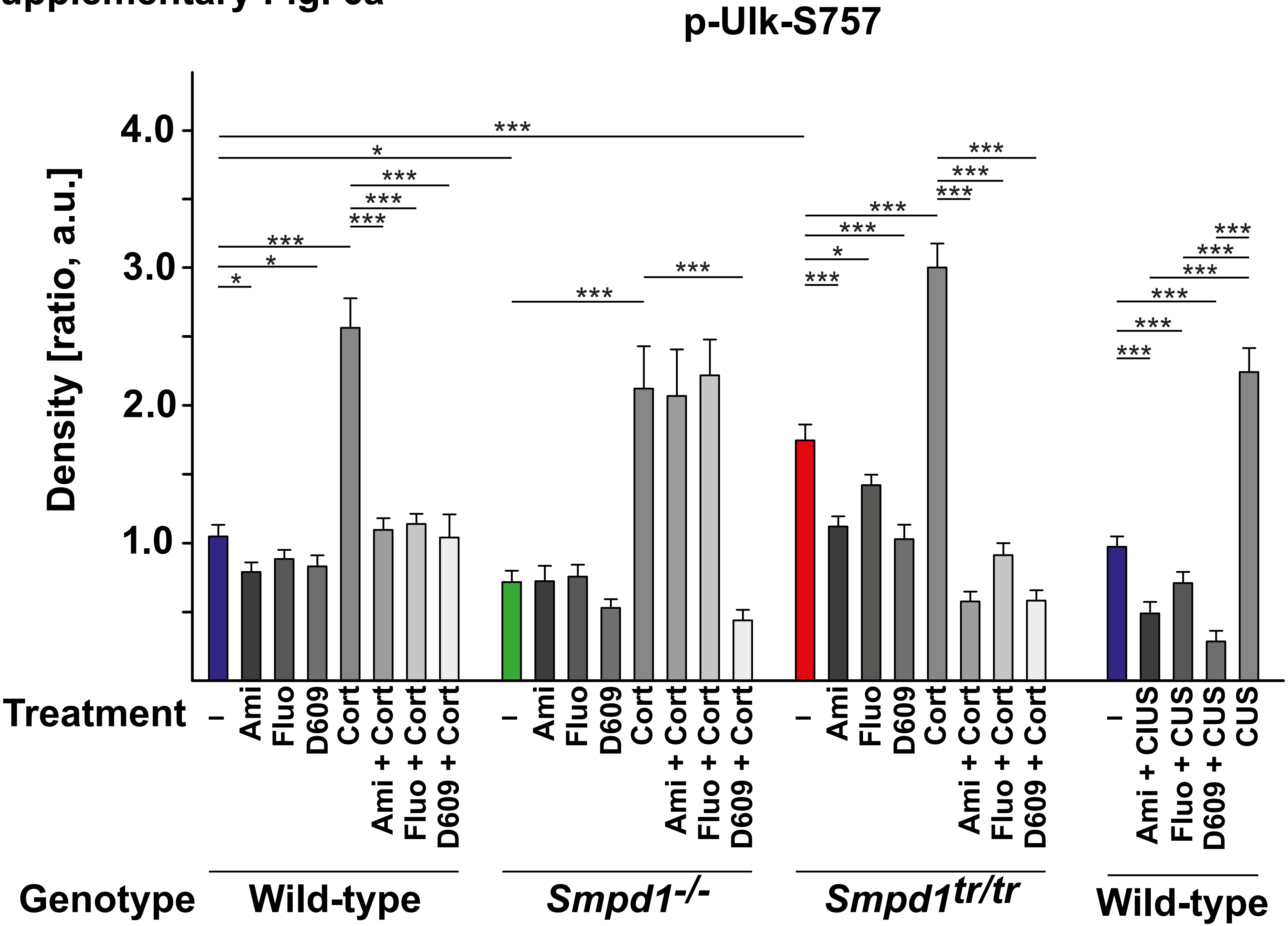

Supplementary Fig. 6b

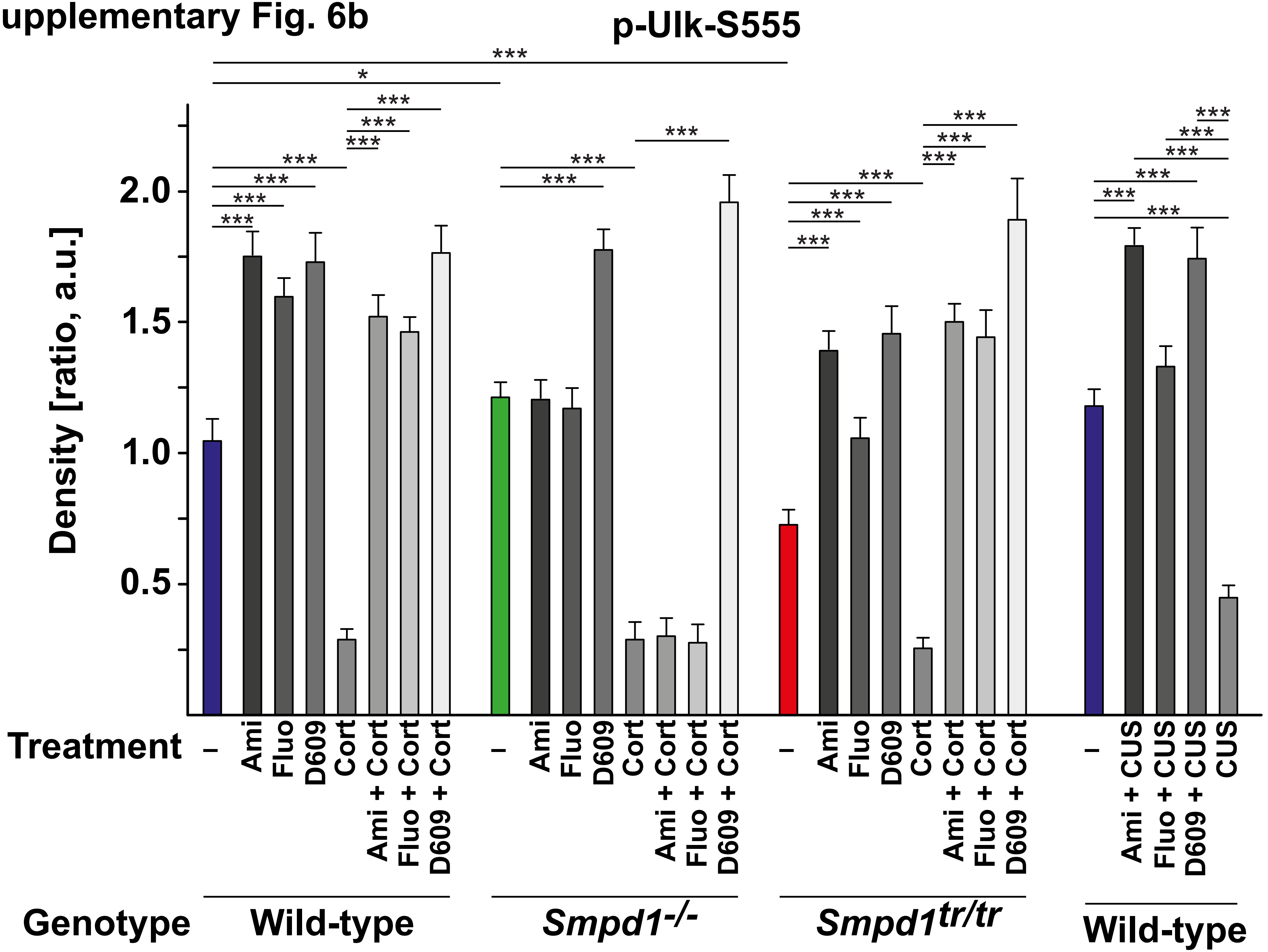

Supplementary Fig. 6c

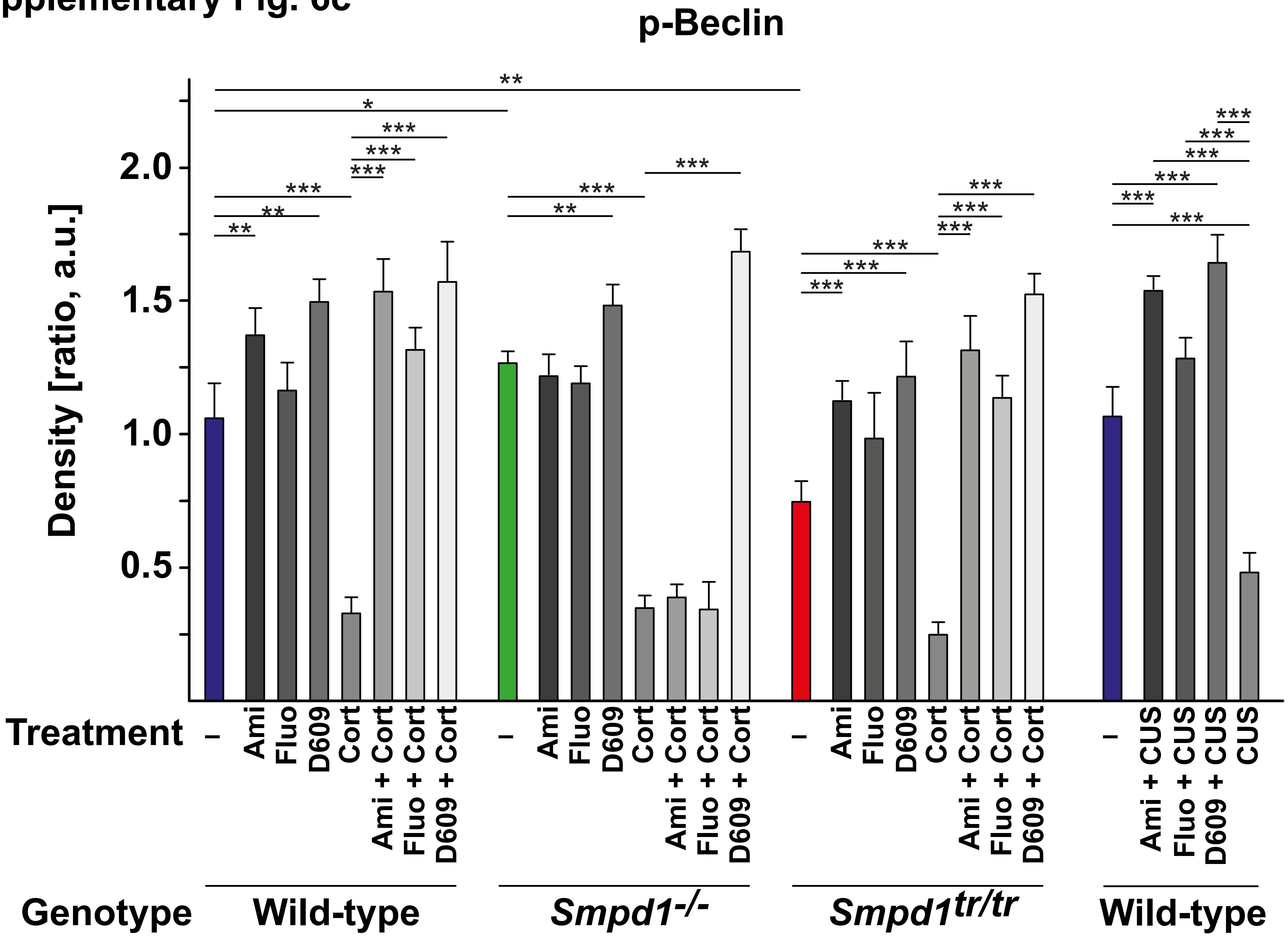

Supplementary Fig. 6d

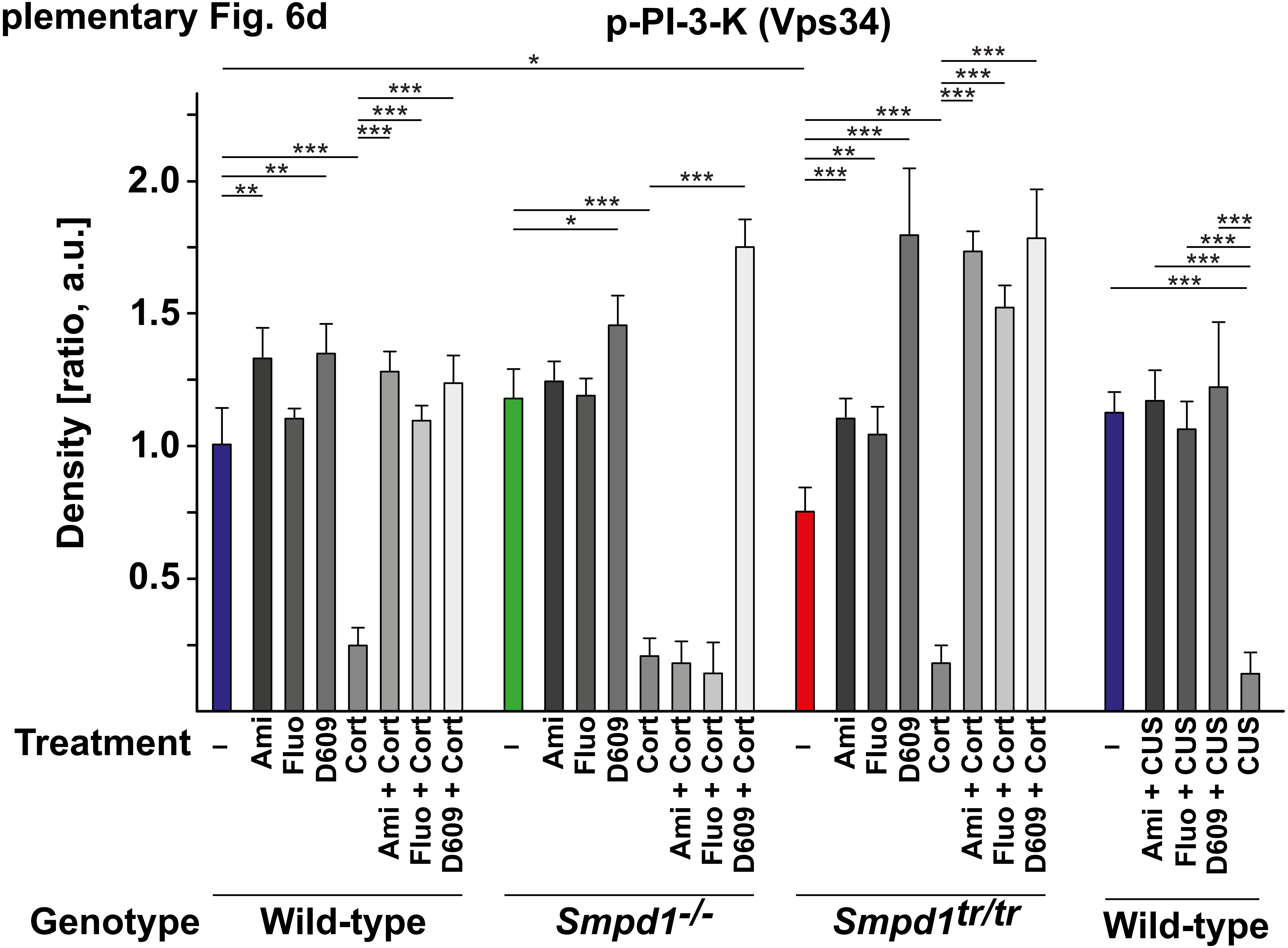

Supplementary Fig. 6e

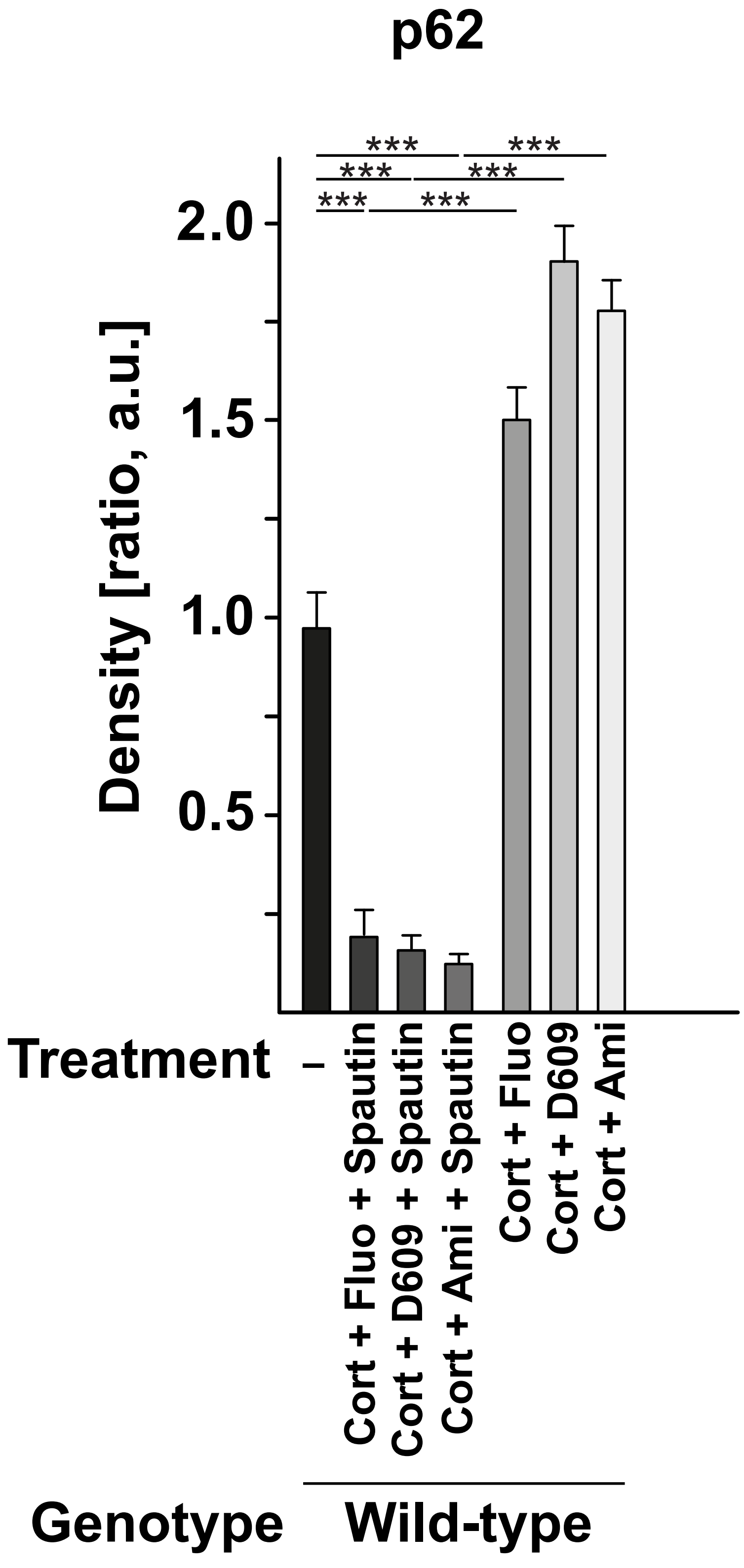

Supplementary Fig. 6f

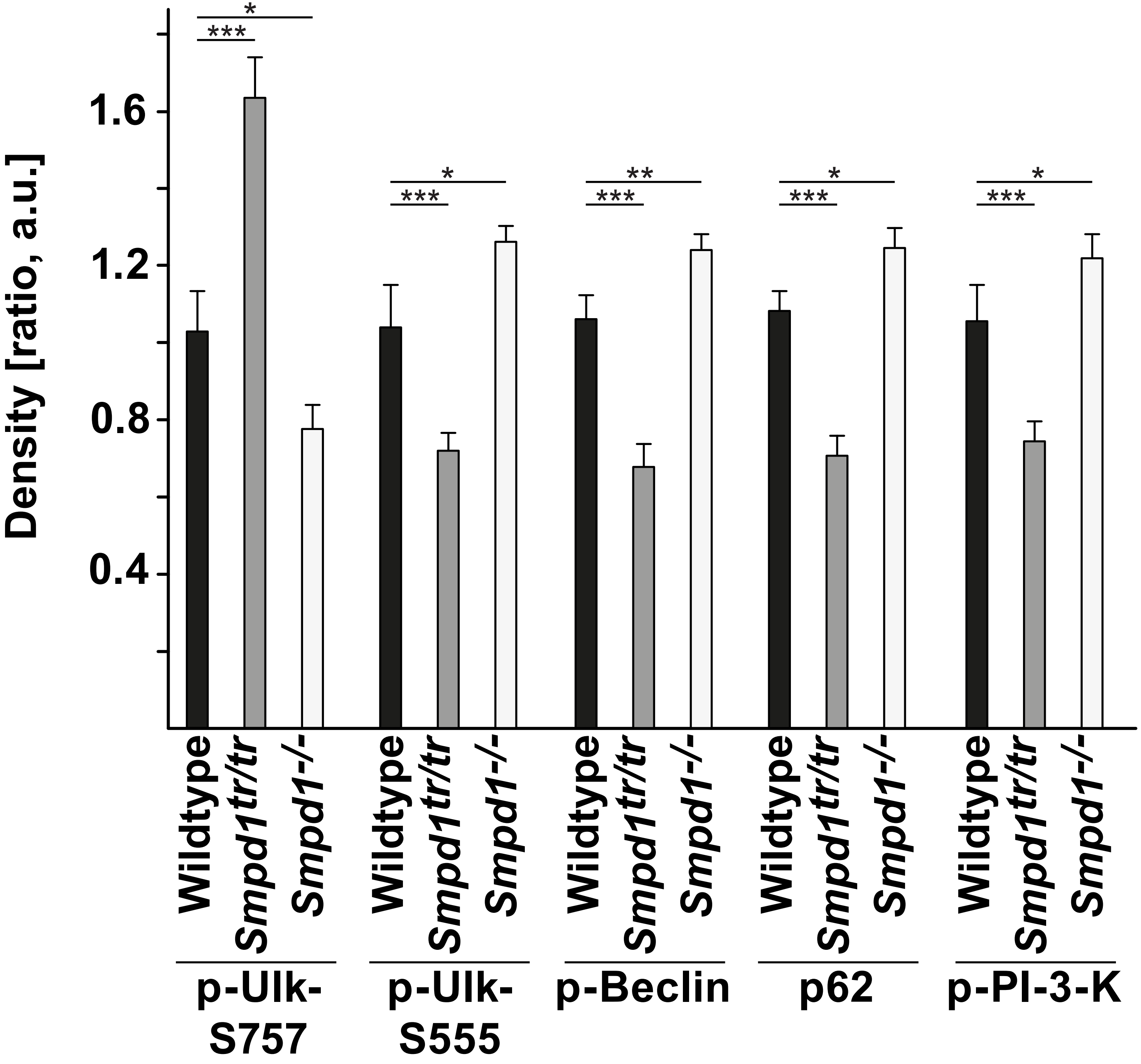

Supplementary Fig. 7

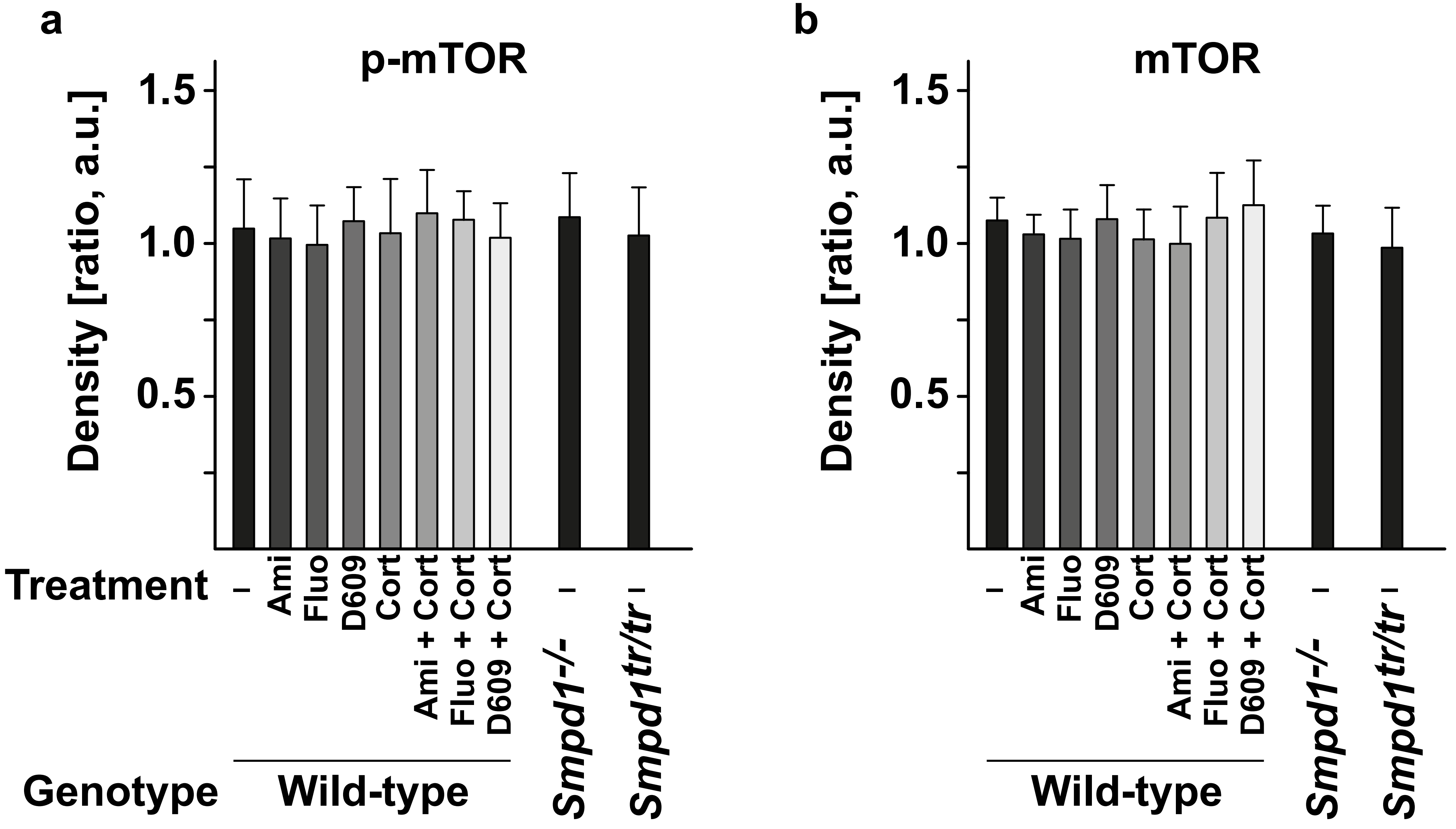

Supplementary Fig. 8a

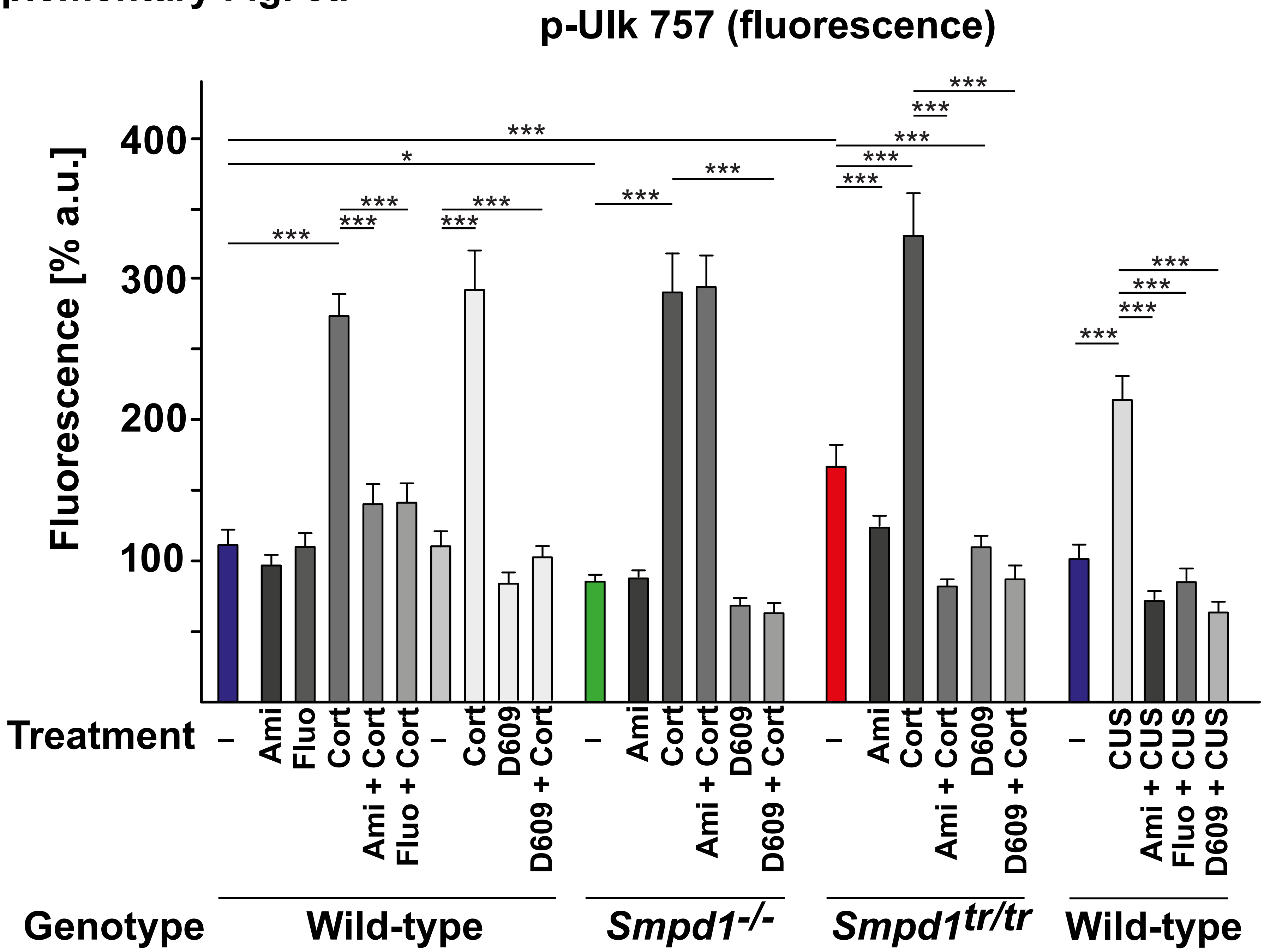

Supplementary Fig. 8b

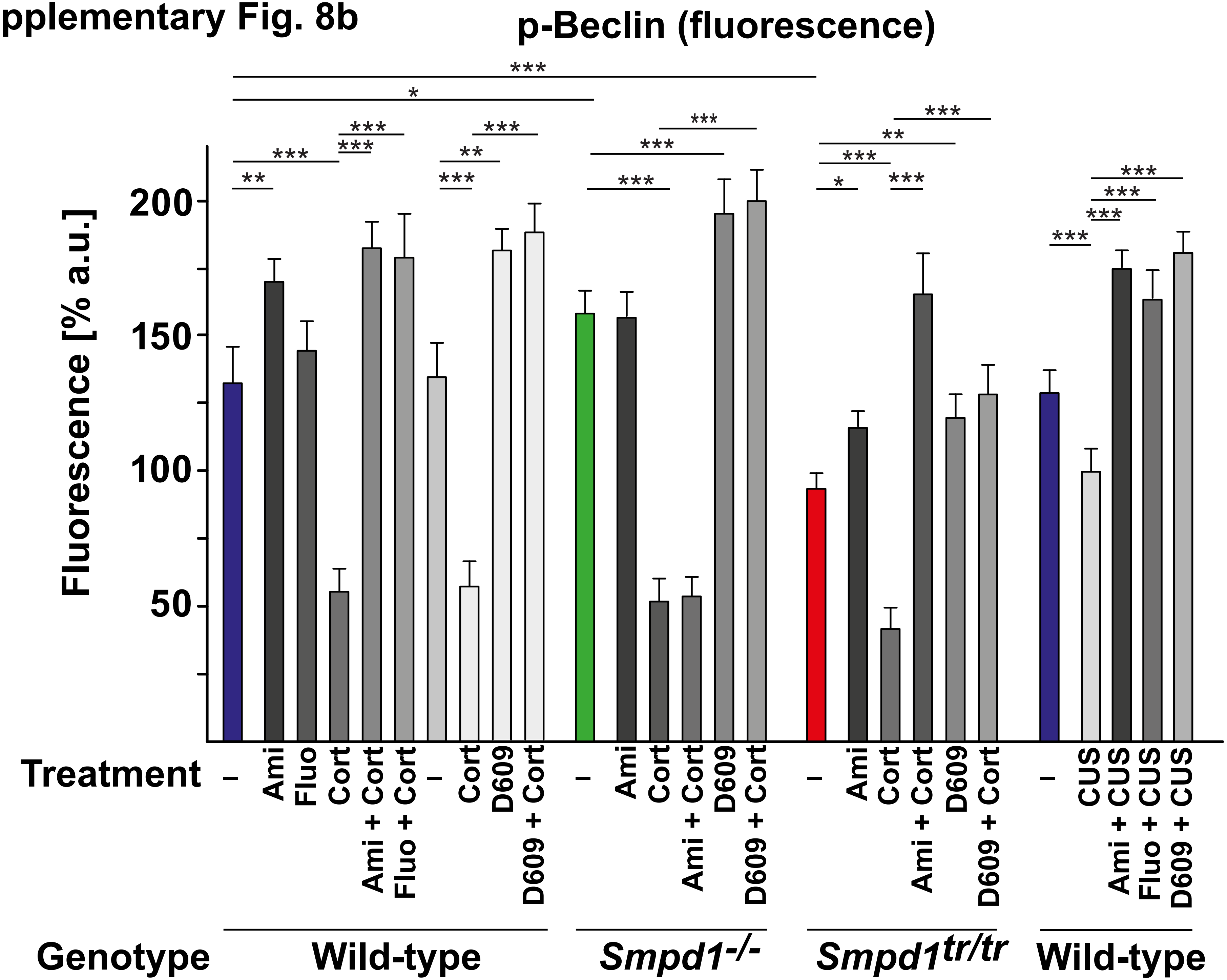

Supplementary Fig. 8c

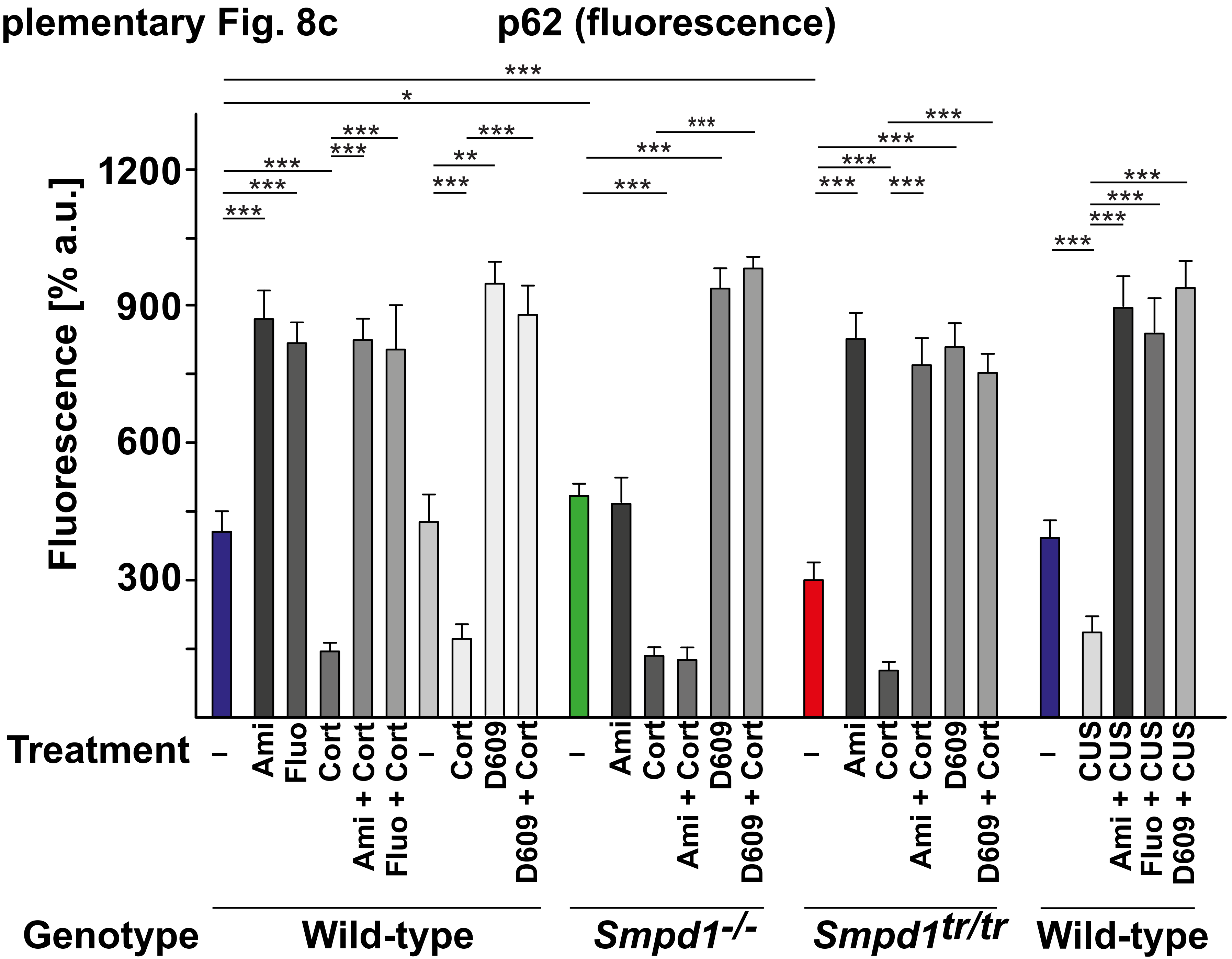

Supplementary Fig. 8d

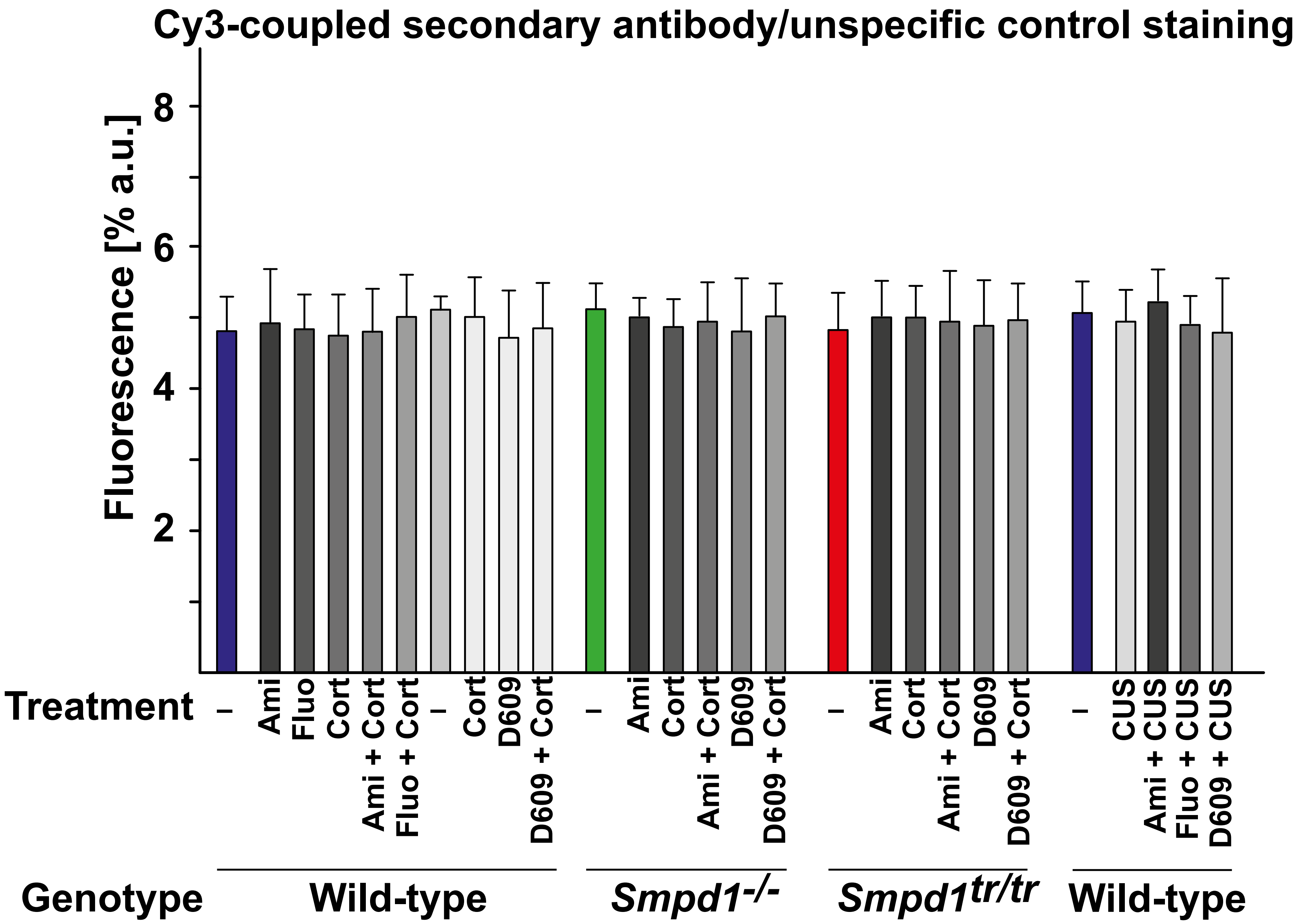

Supplementary Fig. 9

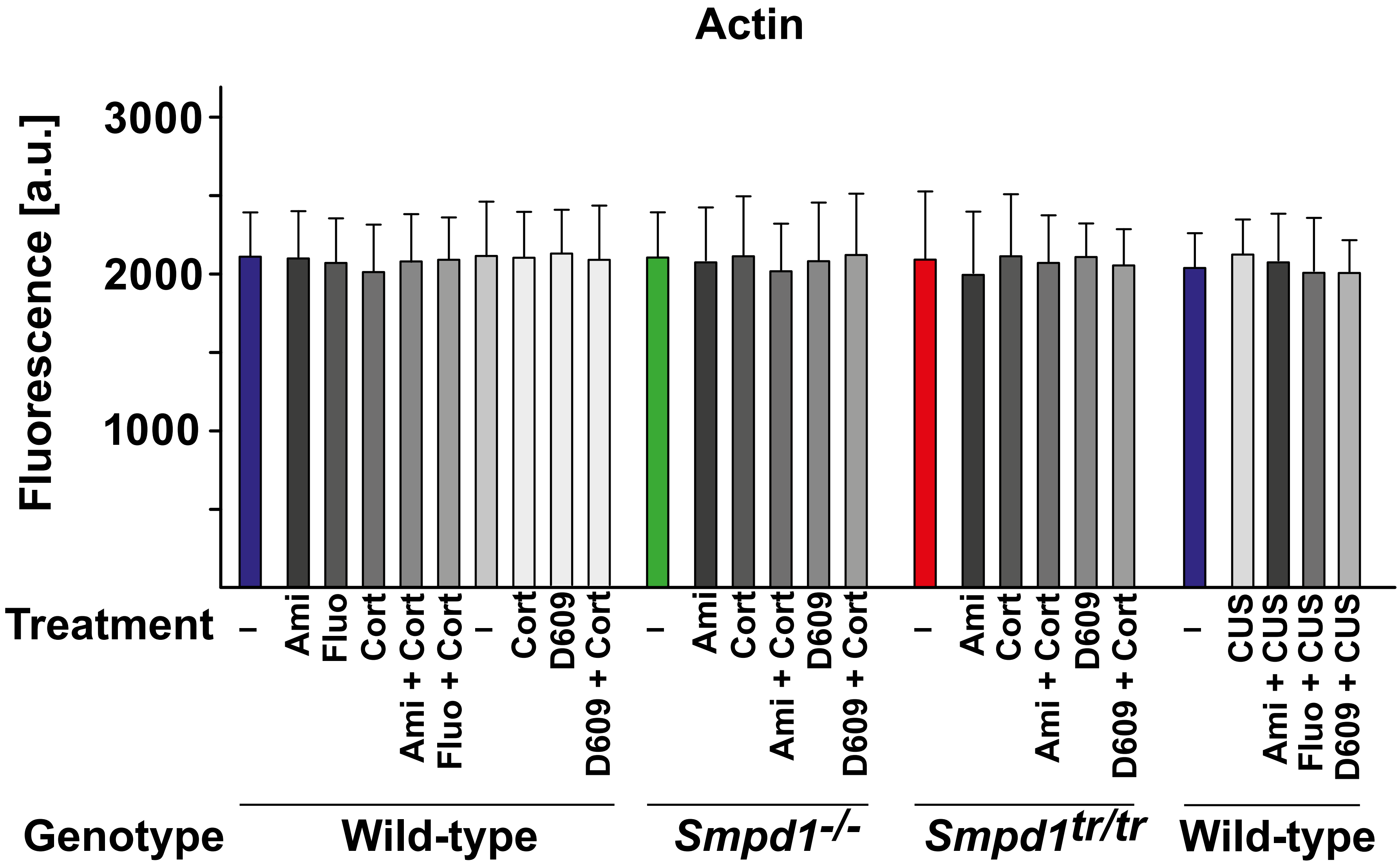

Supplementary Fig. 10a

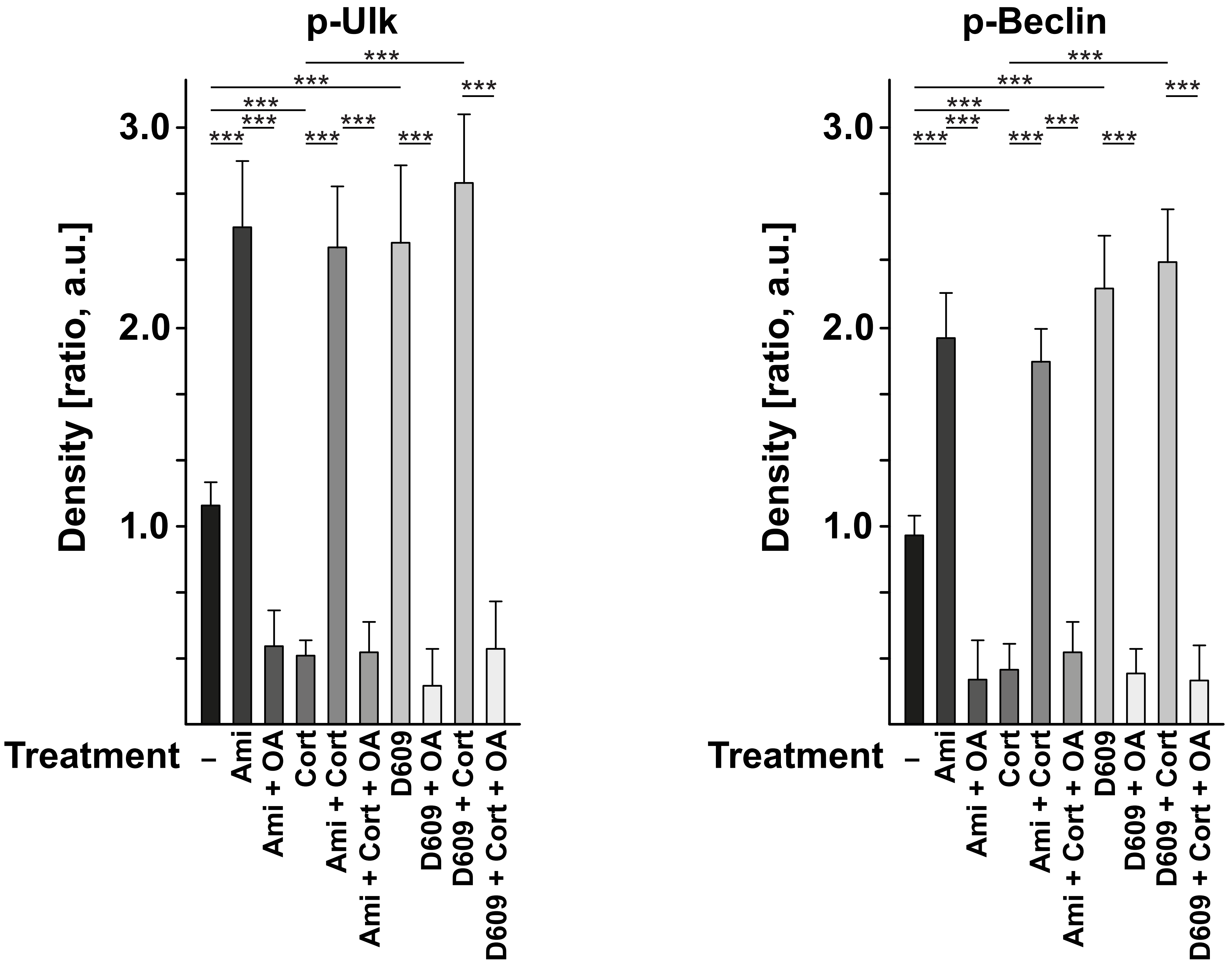

Supplementary Fig. 10b

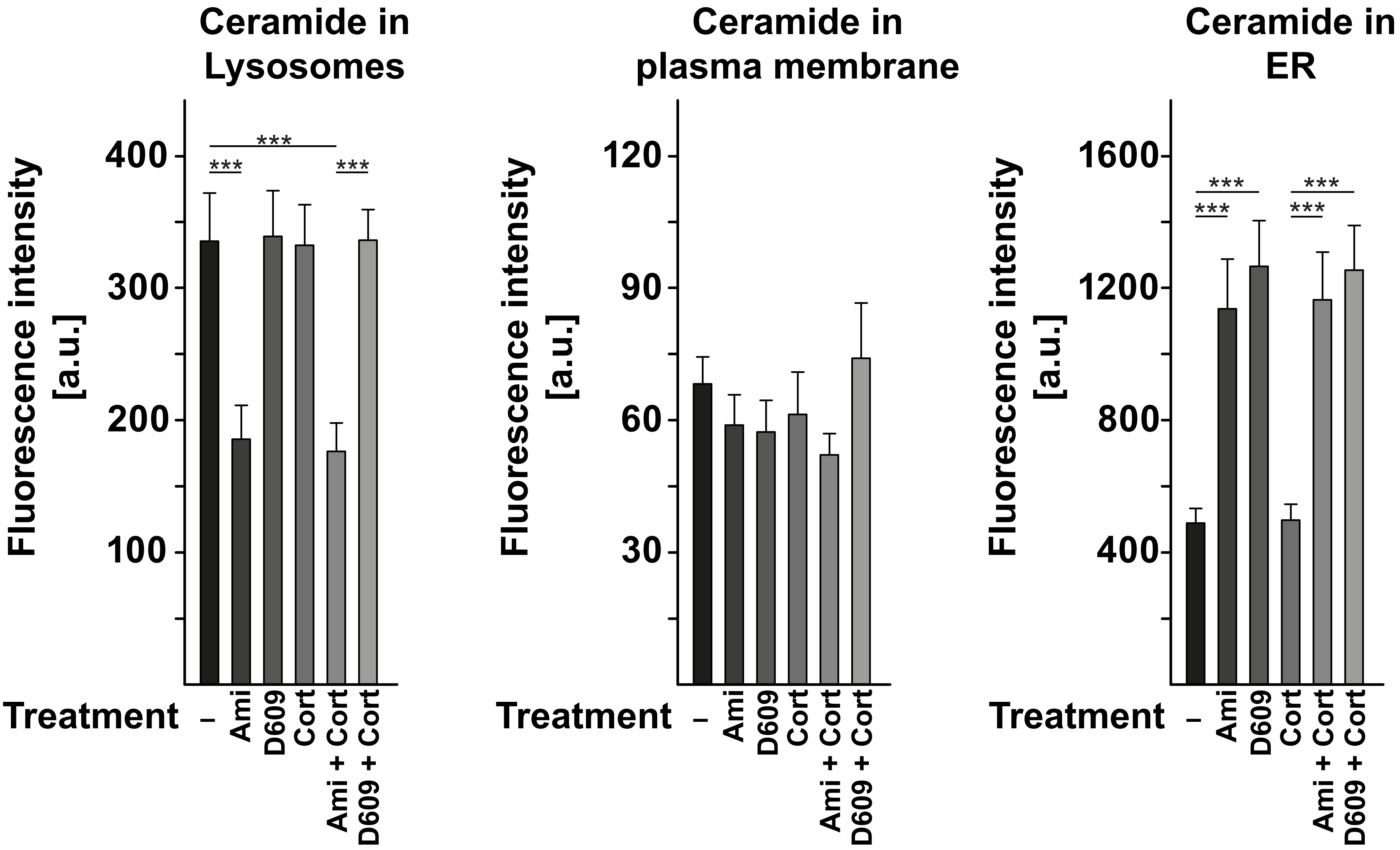

Supplementary Fig. 10c

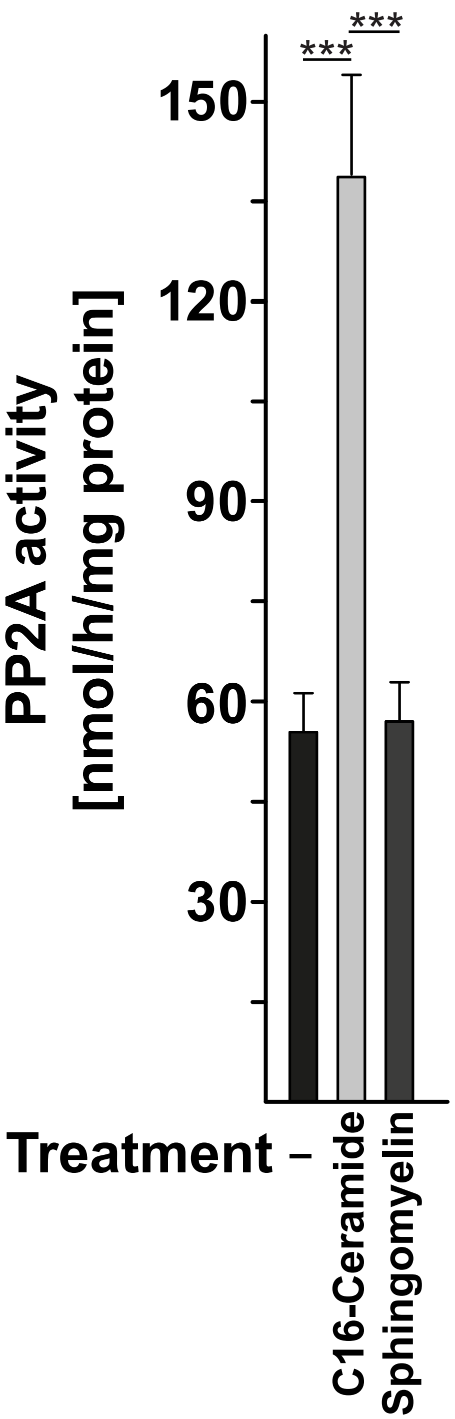

Supplementary Fig. 11

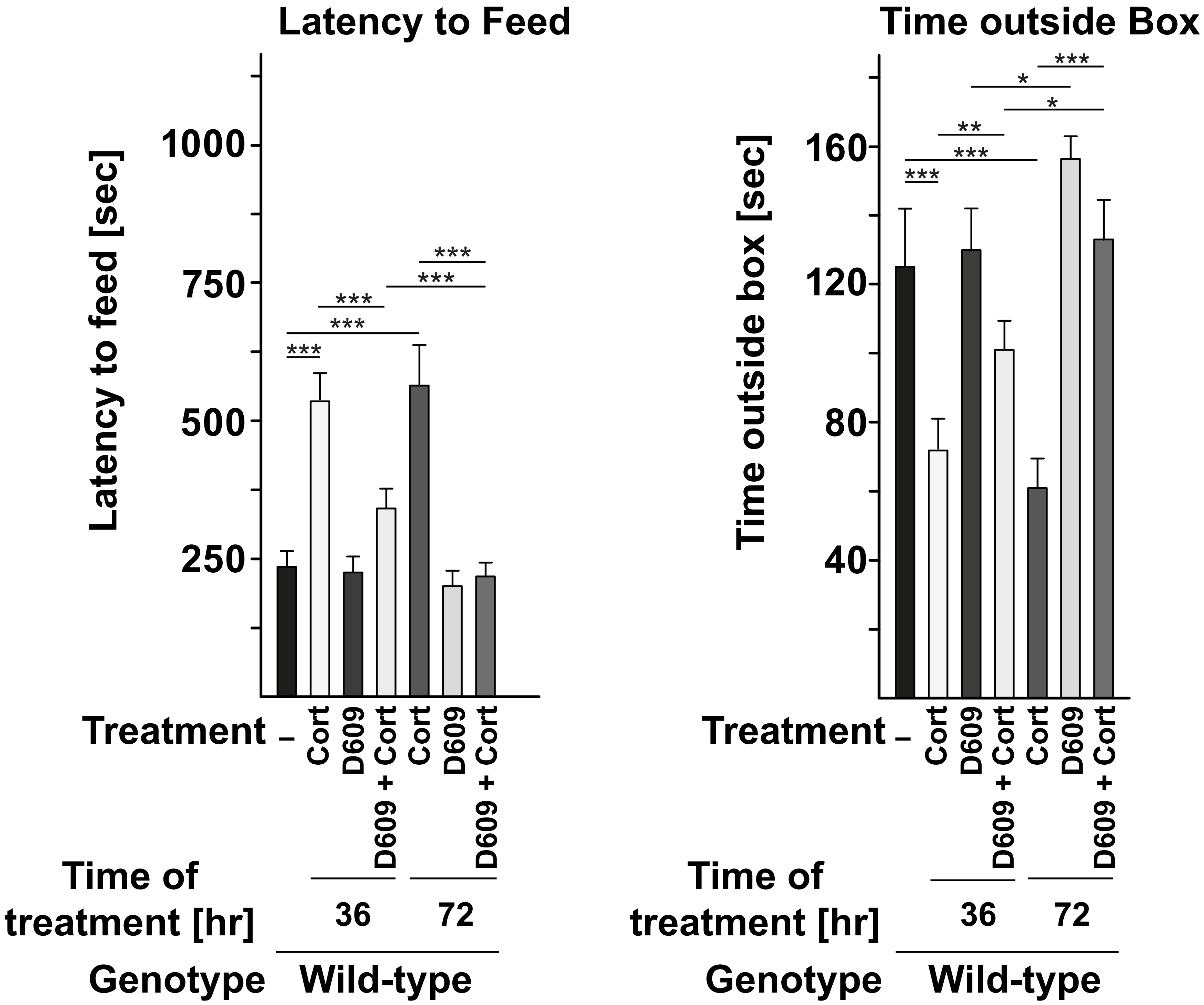

Supplement: Supplementary file 3 — Supplementary Figures [file 41380_2018_90_MOESM3_ESM.pdf]
